# Codon adaptation by synonymous mutations impacts the functional properties of the estrogen receptor‐alpha protein in breast cancer cells

**DOI:** 10.1002/1878-0261.13399

**Published:** 2023-03-01

**Authors:** Léa Clusan, Frederic Percevault, Emmanuelle Jullion, Pascale Le Goff, Christophe Tiffoche, Tamara Fernandez‐Calero, Raphaël Métivier, Monica Marin, Farzad Pakdel, Denis Michel, Gilles Flouriot

**Affiliations:** ^1^ Univ Rennes, Inserm, EHESP, Irset (Institut de Recherche en Santé, Environnement et Travail), UMR_S1085 France; ^2^ Institut de Génétique De Rennes (IGDR), UMR 6290 CNRS, ERL INSERM U1305 Univ Rennes France; ^3^ Departamento de Ciencias Exactas Y Naturales Universidad Catolica del Uruguay Montevideo Uruguay; ^4^ Bioinformatics Unit Institut Pasteur Montevideo Uruguay; ^5^ Biochemistry‐Molecular Biology, Facultad de Ciencias Universidad de la República Montevideo Uruguay

**Keywords:** breast cancer, codon usage, co‐translational folding, endocrine resistance, oestrogen receptor alpha

## Abstract

Oestrogen receptor‐alpha (ERα) positivity is intimately associated with the development of hormone‐dependent breast cancers. A major challenge in the treatment of these cancers is to understand and overcome the mechanisms of endocrine resistance. Recently, two distinct translation programmes using specific transfer RNA (tRNA) repertoires and codon usage frequencies were evidenced during cell proliferation and differentiation. Considering the phenotype switch of cancer cells to more proliferating and less‐differentiated states, we can speculate that the changes in the tRNA pool and codon usage that likely occur make the ERα coding sequence no longer adapted, impacting translational rate, co‐translational folding and the resulting functional properties of the protein. To verify this hypothesis, we generated an ERα synonymous coding sequence whose codon usage was optimized to the frequencies observed in genes expressed specifically in proliferating cells and then investigated the functional properties of the encoded receptor. We demonstrate that such a codon adaptation restores ERα activities to levels observed in differentiated cells, including: (a) an enhanced contribution exerted by transactivation function 1 (AF1) in ERα transcriptional activity; (b) enhanced interactions with nuclear receptor corepressor 1 and 2 [NCoR1 and NCoR2 (also known as SMRT) respectively], promoting repressive capability; and (c) reduced interactions with SRC proto‐oncogene, non‐receptor tyrosine kinase (Src) and phosphoinositide 3‐kinase (PI3K) p85 kinases, inhibiting MAPK and AKT signalling pathway.

## Graphical abstract

This work shows that changing the codons used to produce the oestrogen receptor‐alpha (ERα) protein to synonymous codons more frequently found in genes specifically expressed in proliferative cells leads to functional changes in the receptor, resulting from modifications in its conformation.

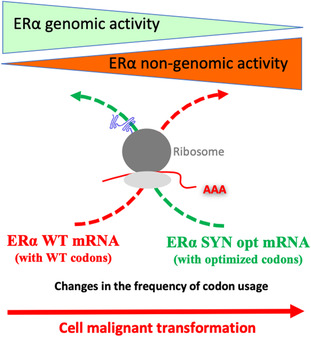

**Abbreviations**
AFs: transactivation functions
AKT: protein kinase B
AP1: activating protein 1
βgal: β‐galactosidase
C3: complement C3
cDNA: complementary DNA
CMV: cytomegalovirus
DMEM: Dulbecco's modified Eagle's medium
E2: 17β‐estradiol
EGF: epidermal growth factor
EGFR: EGF receptor
EMT: epithelial–mesenchymal transition
ERα: oestrogen receptor alpha
ERE: ERα‐responsive element
ERK: extracellular signal‐regulated kinases
FBS: foetal bovine serum
GFP: green fluorescent protein
ICI: ICI 182–780
Luc: luciferase
MAPK: mitogen‐activated kinases
MRTFA: myocardin‐related transcription factor A
MSK1: mitogen and stress‐activated kinase 1
NCoR1: nuclear receptor corepressor 1
NcoR2: nuclear receptor corepressor 2
OHT: 4‐hydroxytamoxifen
ONPG: O‐nitrophenyl β‐D‐galactopyranoside
PBS: phosphate buffered saline
PI3K: phosphoinositide 3‐kinase
PLA: proximity ligation assay
SERD: selective receptor degrader
SERM: selective oestrogen receptor modulators
SP1: specific protein 1
Src: SRC proto‐oncogene, non‐receptor tyrosine kinase
SRC1: steroid receptor coactivator 1
SRC3: steroid receptor coactivator 3
SYN opt: synonymous optimized
tk: thymidine kinase
tRNA: transfer RNA
TUNEL: terminal deoxynucleotidyl transferase dUTP nick end labelling
WT: wildtype

## 1 Introduction

As the main mediator of oestrogens, the oestrogen receptor‐alpha (ERα) is involved in a variety of physiological processes ranging from the establishment and maintenance of the female sexual differentiation patterns to cardiovascular and neuronal systems, to liver, fat and bone metabolisms [1, 2]. ERα influences also several pathological processes of which the most common is breast cancer [3, 4]. A majority of studies show that ERα is absent or expressed at very low level in breast cancer stem cells and instead appears in breast cancers derived from luminal cells [5, 6]. Approximatively 70% of diagnosed breast cancers express ERα whose activation by oestrogens favours the proliferation of breast cancer cells. For these cancers, the therapy of choice is endocrine therapy, which aims to deprive the tumour of oestrogens or directly block ERα activity, respectively, through the use of aromatase inhibitors or antioestrogens such as the tamoxifen [7, 8]. However, endocrine resistance occurs in 30% of cases due to a hormonal escape, with cells continuing to proliferate without oestrogenic stimulation and becoming resistant to endocrine therapy. Breast tumours then switch to less‐differentiated phenotypes and undergo invasive and metastatic processes associated with unfavourable vital prognosis [7].

ERα belongs to the nuclear receptor superfamily, and like all members, it is primarily a transcription factor that exhibits genomic activity through the regulation of gene expression. ERα transcriptional activity is mediated by two transactivation functions (AF): AF1, localized in the N‐terminal part of the protein, and AF2 in the C‐terminal ligand‐binding domain. These two functional domains present interface surfaces that allow the recruitment of coactivators or corepressors in an ordered, cyclical and combinatorial manner [9, 10]. Both AFs do not participate equally in ERα genomic activity, and it has been showed that their contribution depends on the cell differentiation stage [11]. The loss of AF1 activity in dedifferentiated cells has notably been linked to the loss of cell–cell junctions, which particularly occurs during epithelial mesenchymal transition (EMT) [11, 12]. In addition to its genomic activity, ERα is also known to modulate non‐genomic membrane signalling pathways through a protein pool located at the cytoplasm and/or plasma membrane and capable of interacting with several kinases such as SRC proto‐oncogene, non‐receptor tyrosine kinase (Src) and phosphoinositide 3‐kinase (PI3K) p85 kinases [13]. Activation of the downstream mitogen‐activated kinase (MAPK) and protein kinase B (AKT) signalling pathways ultimately leads to the stimulation of cell proliferation and survival [14].

Several mechanisms are known to promote endocrine resistance. These include ER‐activating mutations, imbalance between coactivators and corepressors expression or overactivation of growth factors signalling pathways [15]. Endocrine resistance is generally accompanied by the epithelial–mesenchymal transition of breast cancer cells and functional modifications of ERα [16, 17]. We recently demonstrated that, during EMT, the activation of the actin/MRTFA pathway results in functional modifications of ERα due to the impairment of its localization and interactions with partners. These mechanisms ultimately lead to a decrease of its transcriptional activity and an increase of its non‐genomic activity, promoting hormonal escape [17, 18]. Such modifications in ERα activity are likely to result from changes in environment and ERα partners as mentioned above but may also be due to protein conformational changes that have an impact on its functional activity. Indeed, ERα is a protein whose multifunctional properties are largely regulated by important and varied allosteric changes in the protein that are provoked either by ligand binding, DNA response element recognition or partner interactions [19, 20, 21, 22]. However, other more upstream mechanisms could also be involved in conformational changes of ERα.

Due to the degeneration of the genetic code, amino acids can be encoded by several codons. These synonymous codons are not equally used in coding sequences, leading to a codon usage bias. A correlation between preferred codons and more abundant transfer RNAs (tRNAs) was observed, suggesting that tRNAs pools and codon usage are essential players in the regulation of gene expression. They may differ depending on the cell type, cell cycle phase or differentiation status of the cell [23, 24]. For instance, Gingold et al. [24] demonstrated in 2014 a strong dependence between tRNA selection and abundance and the transcriptional programmes involved during cell proliferation and differentiation. In particular, the tRNA pool was correlated to codon usage bias in genes specifically expressed in differentiated or proliferating cells improving expression of cell state specific genes. More recently, specific expression of tRNAs in metastatic breast cancer cells that may promote tumour progression has been observed [25]. In fact, the availability of tRNA appears to be a key parameter impacting translation speed, and translation speed modification can alter the co‐translational folding of some proteins [26, 27, 28, 29]. Concerning ERα, previous work notably suggested that its conformation would be sensitive to the cellular environment and codon composition [30, 31]. It should be recalled that in healthy breast tissues, ERα is expressed in differentiated cells that do not or rarely proliferate, whereas in ERα‐positive breast cancers, it is often expressed in proliferative cells [32]. A conformational change in ERα during tumour transformation or EMT could, therefore, be induced by a change in tRNA abundance and codon usage, altering the translation rate and final conformation of the receptor.

In the present study, we show that ERα codon usage fits the codon usage of genes specifically expressed in differentiated cells. We generated an oestrogen receptor synonymous sequence to adapt the frequency of ERα codon usage to that observed in genes specifically expressed in proliferative cells, and then investigated the functional consequence of these synonymous mutations on ERα activity. We demonstrate that these synonymous mutations improve the AF1 transcriptional activity of ERα and alter ERα interactions with its partners by reducing its interaction with kinases and promoting the formation of complexes with corepressors. Finally, the expression of the synonymous mutant in MCF7 cell line abolishes the anti‐apoptotic effect of oestradiol.

## 2 Materials and methods

### 2.1 Cell culture and treatments

HEK293 (RRID:CVCL_0045; ATCC origin), HepG2 (RRID:CVCL_0027; ATCC origin), MCF7 (RRID:CVCL_0031; ATCC origin), MDA‐MB‐231 (RRID:CVCL_0062; ATCC origin) and SUM159PT (RRID:CVCL_5423; Kuperwasser Laboratory, Boston, MA, USA) cells were routinely maintained in Dulbecco's Modified Eagle's Medium (DMEM; Gibco, ThermoFisher Scientific, Waltham, MA, USA) supplemented with 8% foetal bovine serum (FBS; Biowest, Nuaillé, France) and antibiotics (Gibco) at 37 °C in 5% CO_2_. Control and overexpressing GFP (Green Fluorescent Protein)‐ERα WT or GFP‐ERα SYN‐opt MCF7 clones were obtained by transfecting cells with the pcDNA6/TR plasmid and the corresponding pcDNA4/TO expression vectors (T‐Rex system, Invitrogen, Waltham, MA, USA) using JetPEI^®^ (Polyplus transfection). GFP epitope was fused at the N‐terminal domain of the proteins. The clones were selected in a medium containing 5 μg·mL^−1^ blasticidin and 100 μg·mL^−1^ zeocin (Invitrogen). Individual clones were isolated and grown in a medium containing selective antibiotics to maintain selection pressure. All cell lines were routinely monitored with luminal and basal‐like markers and mycoplasma detection tests. When treatments with steroids were required, the cells were maintained 48 h in DMEM (Gibco) supplemented with 2.5% dextran/charcoal‐stripped FBS (dsFBS; Biowest) prior to the experiments. The induction of GFP‐ERα WT or GFP‐ERα SYN‐opt expression in MCF7 clones was performed with 1 μg·mL^−1^ tetracycline for 48 h. Pharmacological treatments were performed with final concentrations of 10 nm 17β‐estradiol (E2), 1000 nm 4‐hydroxytamoxifen (OHT), 100 nm ICI 182–780 (ICI) or the solvent 0.1% ethanol as a control. EGF was used at 50 ng·mL^−1^. Cycloheximide was used at 20 μg·mL^−1^.

### 2.2 Plasmids and transient transfections

The ERE (ERα‐responsive element)‐tk (thymidine kinase gene promoter)‐Luc (luciferase), C3 (complement C3 gene promoter)‐Luc, AP1 (activating protein 1 gene promoter)‐Luc, SP1 (specific protein 1 gene promoter)‐Luc and pTAL‐Luc reporter genes as well as the internal control CMV (cytomegalovirus promoter)‐βgal (β‐galactosidase), the pCR‐ERα, pCR‐ERα Δ79, pCR‐ERα Δ173, pCR‐GFP, pCR‐SRC1 (Steroid receptor coactivator 1) and pCR‐NCoR1 (nuclear receptor corepressor 1) expression vectors have been previously described [12, 33, 34]. pCR‐ERα SYN‐opt was produced synthetically (GeneArt, Life Technologies, ThermoFisher Scientific, Waltham, MA, USA). pCR‐ERα SYN‐opt Δ79 and pCR‐ERα SYN‐opt Δ173 expression vectors were generated by subcloning corresponding PCR products into pCR3.1 vector (Invitrogen). To measure ERα mRNA expression level in transient transfection experiments, 500 ng of expression vectors by well was transfected in 6‐well plates together with 500 ng of pTAL‐LUC used as an internal control. To assess ERα transcriptional activity on reporter genes, 50 ng of control (pCR3.1) or ERα expression vectors were co‐transfected with 200 ng of reporter genes and 200 ng CMV‐βgal (β‐galactosidase) by well in 24‐well plates. When pCR‐MRTFA ΔN200, pCR‐SRC1 or pCR‐NCoR1 expression vectors were added to the DNA mix, 200 ng of plasmid was used. For immunofluorescence and proximity ligation assay, 250 ng of expression vectors was used by well in 24‐well plates. For western blot analysis, 500 ng of expression vectors was transfected by well in 6‐well plates. Transfections were carried out with JetPEI^®^ (Polyplus transfection) according to the manufacturer's instructions. GFP‐ERα WT or GFP‐ERα SYN‐opt expression vectors were generated from the pcDNA4/TO expression vector (T‐Rex system, Invitrogen) by subcloning the corresponding PCR products.

### 2.3 Luciferase assay

Cells were plated in 24‐well plates and incubated for 24 h before medium exchange for serum and steroid starvation. Cells were transfected as previously described and treated with ligands for 24 h before luciferase assay (Luciferase Assay System, Promega, Madison, WI, USA) [35]. β‐galactosidase activity was determined by incubating two third of each lysate with 0.7 mg·mL^−1^ O‐nitrophenyl β‐D‐galactopyranoside (ONPG) in assay buffer (0.8 mm MgCl_2_, 35 mm β‐mercaptoethanol) before measuring the absorbance at 415 nm with an iMark Microplate Absorbance Reader (Bio‐Rad, Hercules, CA, USA). Luciferase activity reflecting ERα transcriptional activity was normalized to the β‐galactosidase activity.

### 2.4 Immunofluorescence and proximity ligation assay (PLA)

Cells were plated on cover slides in 24‐well plates and incubated for 24 h before medium exchange for serum and steroid starvation. When cell treatment was completed, phosphate buffered saline (PBS) containing 4% paraformaldehyde was used to fix the cells for 15 min. After PBS washing, cells were permeabilized in PBS containing 0.3% Triton X‐100 for 15 min. After washing, an incubation with primary antibodies (1 : 1000) in PBS containing 3% FBS was performed overnight at 4 °C. For immunofluorescence experiments, cells were incubated the next day with secondary antibodies (1 : 1000) in PBS‐FBS for 2 h at room temperature. For PLA, the Duolink^®^ Proximity Ligation Assay reagents from Sigma‐Aldrich (Saint Louis, MO, USA) were used to detect ERα interactions with partners, according to manufacturer's instructions, as previously described [17]. Finally, the cover slides were mounted in Duolink^®^ *In Situ* Mounting Medium with DAPI (Sigma‐Aldrich). Images were obtained with an ApoTome Axio Z1 Imager microscope (Zeiss, Göttingen, Germany) and processed with axiovision software (Zeiss). Fluorescent cells and PLA dots were analysed using imagej software.

### 2.5 Western blotting

Whole‐cell extracts were lysed in RIPA buffer or directly prepared in 3× Laemmli buffer from subconfluent cells in 6‐well plates. Subcellar fractionation of HepG2 cells was performed as previously described [17]. Following sonication, the protein extracts were denatured for 5 min at 95 °C, separated on 10% SDS polyacrylamide gels and transferred to nitrocellulose membrane (Amersham Biosciences, Amersham, UK). The proteins were then probed with specific antibodies (1 : 1000) as previously described [11] and detected using the Substrat HRP Immobilon Western kit from Millipore (Burlington, MA, USA).

### 2.6 Proliferation assay

Cells were plated in 24‐well plates and incubated for 24 h before medium exchange for serum and steroids starvation. Tetracycline induction and pharmacological treatments were applied for 6 days before cell trypsinization and counting.

### 2.7 Flow cytometry analysis

Cells were plated in 10‐cm dishes and incubated for 24 h before medium exchange for serum and steroids starvation. After 48 h tetracycline induction and pharmacological treatments, cells were collected in PBS containing 30% IFA buffer (10 mm HEPES, pH 7.4, 150 mm NaCl, 4% FBS) and centrifuged for 10 min at 800 ***g***, 4 °C. Cells were fixed for 30 min on ice with 70% ethanol and incubated for 20 min in IFA buffer at 4 °C. A 30‐min incubation with 100 μg·mL^−1^ RNAse A was then performed at 37 °C, and 50 μg·mL^−1^ propidium iodide (Sigma) was finally added for 10 min at 37 °C. Finally, the cell cycle was then analysed using the FACSCalibur flow cytometer (BD Biosciences, Franklin lakes, HI, USA).

### 2.8 TUNEL staining

Apoptosis was determined by detecting DNA fragmentation using terminal deoxynucleotidyl transferase dUTP nick end labelling (TUNEL) staining. This was performed with an *In Situ* Cell Death Detection Kit, Fluorescein (Roche, Basle, Switzerland) according to the manufacturer's instructions, as previously described [34]. Images were obtained with an ApoTome Axio Z1 Imager microscope (Zeiss) and processed with axiovision software. TUNEL‐positive cells were analysed using imagej software.

### 2.9 Quantitative RT‐PCR (RT‐qPCR)

For transient transfection experiments, cells were plated in 6‐well plates, and 24 h later the medium was replaced with medium containing 2.5% dextran/charcoal‐stripped FBS before transient transfection. RNA was extracted 48 h after transfection. For stable cell lines, cells were plated in 6‐well plates and incubated for 24 h before medium exchange for serum and steroids starvation. RNA was extracted after 48 h tetracycline induction and 24 h pharmacological treatments. RNA extraction was performed using the Nucleospin RNA Plus kit (Macherey‐Nagel, Düren, Germany) according to manufacturer's instruction. Quantitative RT‐PCR was performed as previously described [34], with the primers sequences indicated in Table S1. Measurement of control (empty), ERα WT and ERα SYN‐opt mRNA expression in transient transfection experiments was performed using primers designed in the common 3′UTR BGH sequence of the plasmid. Results were normalized to pTAL‐Luc mRNA expression after subtraction of non‐reverse transcribed products. mRNA expressions from stable cell lines were normalized to TBP mRNA level.

### 2.10 Protein degradation and limited digestion

Cells were plated in 6‐well plates and incubated for 24 h before medium exchange for serum and steroids starvation. Cells were then treated 48 h with tetracycline. Analysis of ERα WT and SYN‐opt protein degradation was performed at different times after cycloheximide treatment. For limited digestion experiments, cells were scrapped in PBS and centrifuged for 7 min at 800 ***g***, 4 °C after 48 h tetracycline induction. Cell pellet was sonicated and the lysate recovered after a 15‐min centrifugation at 12 000 ***g***, 4 °C. After protein quantitation with the DC™ Protein Assay kit (Bio‐Rad), a same amount of proteins was digested by 0.2–1.6 ng·μL^−1^ trypsin or chymotrypsin (Sigma Aldrich) for 30 min at 30 °C. Enzymatic reaction was stopped by adding 1× Laemmli buffer, and the samples were denatured for 5 min at 95 °C before western blotting.

### 2.11 Reagents and antibodies

17β‐estradiol (E2), 4‐hydroxy‐tamoxifen (OHT) and cycloheximide were purchased from Sigma‐Aldrich. ICI 182–780 (ICI) was obtained from TOCRIS Bioscience (Bristol, UK). The primary antibodies used for western blotting and immunofluorescence analyses were as follows: antibodies from Santa Cruz Biotechnology (Santa Cruz, CA, USA) against C‐term ER (HC‐20, sc‐543), p‐Akt1/2/3 (Ser473; sc‐7985‐R), Akt1/2/3 (H‐136, sc‐8312), ERK1 (K‐23, sc‐94) and p‐ERK (E‐4, sc‐7383); antibodies from Abcam (Cambridge, UK) against H3S10p (ab5176), H3 (ab12079) and Lamin A + C (JOL2, ab40567); antibody from ThermoFisher Scientific against N‐term ER (6F11, MA5‐13304); antibody from Becton Dickinson (Franklin lakes, HI, USA) against GFP (JL‐8). The secondary peroxidase‐conjugated donkey anti‐rabbit (NA934V) and sheep anti‐mouse (NA931V) antibodies were purchased from GE Healthcare (Chicago, IL, USA). Alexa Fluor^®^ dye‐conjugated secondary antibodies from Invitrogen were employed for immunofluorescence.

### 2.12 Bioinformatic analysis

Regulatory elements recognition and promoter and enhancer prediction were performed with Nsite and FPROM respectively [36, 37]. Default parameters were used in both cases. Prediction of secondary structures of mRNA was performed with RNAfolder [38].

### 2.13 Statistical analysis

Statistical analyses were performed using Student's *t*‐test. The values are provided as the mean ± standard error of the mean (SEM).

## 3 Results

### 3.1 ERα codon usage is adapted to the translation programme of differentiated cells and less adapted to the translation programme of proliferating cells

**Fig. 1:**
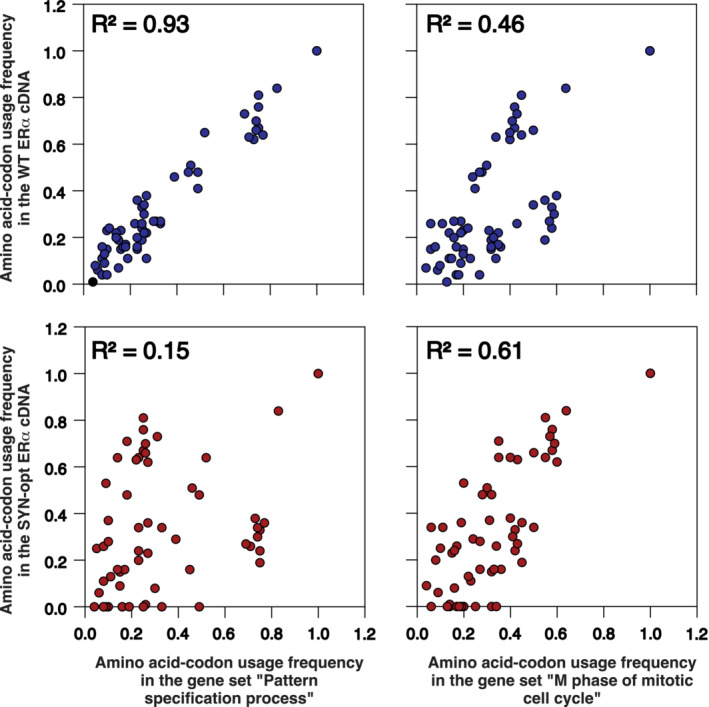
ERα codon usage is adapted to the translation programme of differentiated cells and less adapted to the translation programme of proliferating cells. Comparison of the amino acid codon usage frequency deduced from the coding sequences of the wildtype (WT) and the synonymous optimized (SYN‐opt) oestrogen receptor‐alpha (ERα) with those of the gene sets ‘M phase of mitotic cell cycle’ (proliferation) and ‘pattern specification process’ (differentiation) from the study of Gingold et al. [24]. Each dot corresponds to one codon. SYN‐opt ERα complementary DNA (cDNA) was generated by selecting for each amino acid a codon whose frequency of use in genes specifically expressed in ‘proliferation’ approximated the frequency of the original codon observed in genes specifically expressed in ‘differentiation.’ Codons with a similar frequency of use between ‘proliferation’ and ‘differentiation’ were maintained (see Fig. S1).

In order to measure the adaptability of ERα codon usage to the dual translation programme in cellular proliferation and differentiation [24], we compared the frequency of codon usage deduced from the coding sequence of the human ERα gene with those of the two functional gene sets, ‘M phase of mitotic cell cycle’ and ‘pattern specification process’ (which we will call hereafter ‘proliferation’ and ‘differentiation’) from Gingold et al.'s study [24]. As expected, codon usage frequency for ERα correlated strongly with that of genes specifically expressed in differentiation (*R* ^2^ = 0.93) and weakly with that of genes specifically expressed in proliferation (*R* ^2^ = 0.46; Fig. 1). Since ERα is increasingly expressed in proliferating cells during mammary epithelial cell carcinogenesis, we generated, based on Gingold et al.'s study [24], an ERα complementary DNA (cDNA) in which codons were mutated to synonymous codons in order to optimize the codon usage of ERα to proliferative cells. Specifically, we chose a codon for each amino acid whose frequency of use in genes specifically expressed in proliferation approximates the frequency of the original codon observed in genes specifically expressed in differentiation. Codons with a similar frequency of use between ‘proliferation’ and ‘differentiation’ were maintained. Thus, a total of 82% of the codons (50 of 61) was changed into synonymous codons. This mutant, called ERα SYN‐opt, has an identical amino acid sequence to ERα wildtype (WT), but a different DNA sequence (Fig. 1 and Fig. S1).

**Fig. 2:**
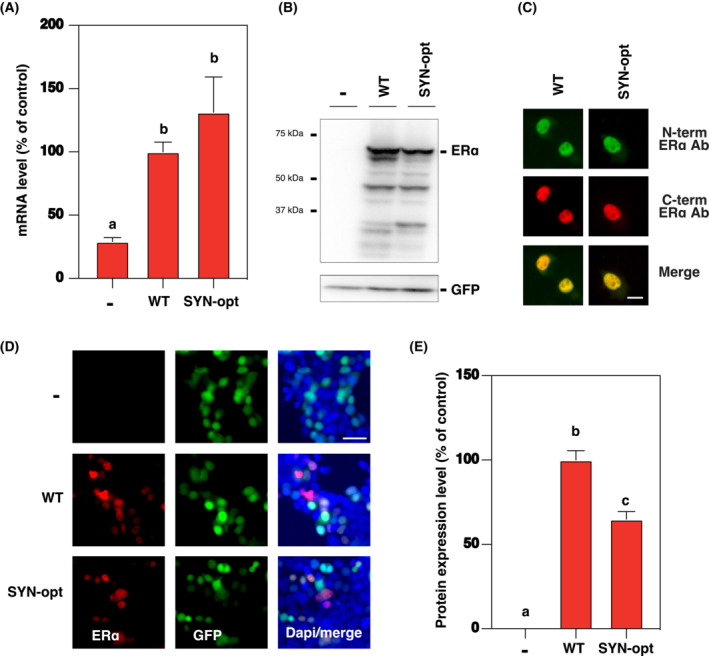
ERα SYN‐opt protein is correctly produced both qualitatively and quantitatively. (A) HEK293 cells were transiently transfected with either empty pCR3.1, pCR‐ERα WT or pCR‐ERα SYN together with pTAL‐luciferase (Luc) reporter genes. And 48 h later, total RNAs were prepared and the expression level of the mRNA transcribed from plasmids was quantified and normalized as described in Section 2. Data correspond to the mean values ± SEM from three experiments and are expressed as a percentage of the expression level of ERα WT mRNA. Columns with the superscripts a and b differ significantly between each superscript (*P* < 0.05, Student's *t*‐test). (B) Western blot analysis of ERα WT and ERα SYN‐opt proteins 48 h after transient transfection of the corresponding expression vectors in HEK293 cells. Green fluorescent protein (GFP) expression of the co‐transfected pCR‐GFP plasmid was used as internal control. The experiment was repeated three times with similar results. (C) Immunofluorescent detection of ERα WT and ERα SYN‐opt proteins by a N‐terminal and C‐terminal ERα antibody (Ab), 48 h after transient transfection of the corresponding expression vectors in HEK293 cells. The experiment was repeated twice with similar results. Scale bar = 10 μm. (D, E) Immunofluorescent detection of ERα and GFP proteins, 48 h after transient transfection of the pCR3.1, pCR‐ERα WT or pCR‐ERα SYN expression vectors together with pCR‐GFP plasmid in HEK293 cells. (D) Representative images are shown. Scale bar = 30 μm. (E) Densitometry quantification of the immunofluorescence images, with identical exposure times, expressed as a percentage of the intensity measured for ERα WT protein and normalized to GFP expression. Results are the means ± SEM. Columns with the superscripts a, b and c differ significantly between each superscript (*n* = 10; *P* < 0.05, Student's *t*‐test).

Changes in the coding sequence can have major consequences in terms of transcriptional and translational activity by possibly creating or altering enhancer sequences, splicing factor binding sites and/or secondary structures of the messenger RNA (mRNA). Therefore, a predictive analysis of DNA regulatory sequences and mRNA secondary structure was first performed on ERα WT and SYN‐opt coding sequences, showing in particular the presence of a TATA box in the coding sequence of ERα SYN‐opt and a slightly more unstable secondary structure of the corresponding mRNA compared with ERα WT (Fig. S2). ERα WT and SYN‐opt mRNA expression levels were then measured after transient transfection of the corresponding pCR expression vectors in HEK293 cells, selected for this study because of their high transfection efficiency. mRNA expression levels were normalized to Luc mRNA produced from a co‐transfected pTAL‐Luc reporter gene and expressed as fold changes from mRNA transcribed from empty pCR3.1 expression vector (Fig. 2A). While the insertion of a coding sequence in the expression vector increases mRNA expression, no significant difference is observed between the expression level of ERα WT and SYN‐opt mRNAs. Translation efficiency of these transcripts was next studied by qualitative and quantitative measuring the translated proteins through Western blot and immunofluorescence experiments. Results were normalized to the expression of the green florescent protein (GFP) produced from a co‐transfected pCR‐GFP plasmid. As illustrated in Fig. 2B–D, ERα SYN‐opt protein is properly expressed, has the right molecular weight and is recognized by antibodies targeting both ERα N‐ and C‐terminal domains. As ERα WT, it displays a major nuclear localization (Fig. 2D). However, its expression level is about 30–40% lower than that of ERα WT. With the ERα SYN‐opt protein correctly produced both qualitatively and quantitively, the functional properties of the synonymous mutant were then studied.

### 3.2 Codon usage optimization of ERα coding sequence by synonymous mutations (ERα SYN‐opt) impacts the behaviours of ERα on ERE‐, AP1‐ and SP1‐driven reporter genes

**Fig. 3:**
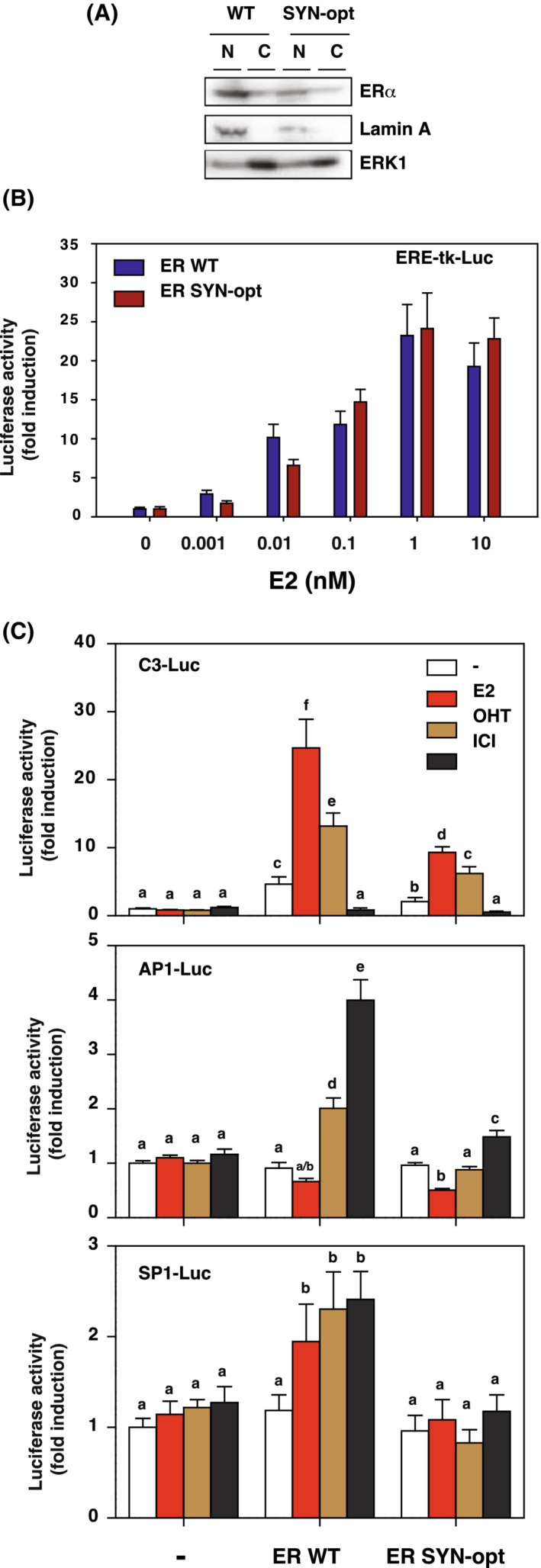
ERα SYN‐opt exhibits different transcriptional activity than ERα WT on ERE‐, AP1‐ and SP1‐driven reporter genes. (A) Western blot analysis of ERα WT and ERα SYN‐opt proteins in nuclear (N) and cytoplasmic (C) fractions of HepG2 cells, 48 h post‐transfection of the corresponding expression vectors. Lamin A expression validates the subcellular fractionation, and extracellular signal‐regulated kinase 1 (ERK1) amounts were used as a loading control. The experiment was repeated twice with similar results. (B) HepG2 cells were transfected with ERE (ERα‐responsive element)‐tk (Thymidine kinase gene promoter)‐Luc and CMV (Cytomegalovirus promoter)‐βgal (β‐Galactosidase) reporter genes together with either pCR ERα WT or pCR ERα SYN‐opt expression vectors and then treated for 24 h with increasing doses of estradiol (E2; 0–10 nm). Luciferase activity was normalized to that of the β‐galactosidase activity. Data correspond to the mean values ± SEM from five separate transfection experiments and are expressed as fold change from non‐treated control. (C) HepG2 cells were transfected with C3 (Complement C3 gene promoter)‐Luc, AP1 (Activating protein 1 gene promoter)‐luc or SP1 (Specific protein 1 gene promoter)‐luc reporter genes together with the CMV‐βgal internal control and either pCR3.1 (empty vector), pCR ERα WT or pCR ERα SYN‐opt expression vectors and then treated for 24 h with E2 (10 nm), 4‐hydroxytamoxifen (OHT; 1 μm), ICI 182–780 (ICI; 100 nm) or vehicle. Luciferase activity was normalized to β‐Galactosidase activity. Data shown are mean values ± SEM from three separate transfection experiments and are expressed as fold change from vehicle‐treated pCR3.1 control. Columns with the superscripts a to f differ significantly between each superscript (*P* < 0.05, Student's *t*‐test).

ERα SYN‐opt transcriptional activity was first characterized following transient transfection in HepG2 cells. This cell line was chosen for its differentiated phenotype, providing an AF1‐permissive context with no endogenous ERα expression, as previously described [11, 12]. As in HEK293 cells, ERα SYN‐opt protein is properly expressed in HepG2 and displays a major nuclear localization with a slight decrease in expression compared to ERα WT protein (Fig. 3A). As ERα WT, the transactivation efficiency of ERα SYN‐opt protein was first assessed on an ERα‐responsive element (ERE)‐driven luciferase reporter gene (ERE‐tk‐Luc) in the presence of increasing E2 concentration. Results show a dose–response curve similar to that observed with ERα WT with an EC50 of approximately 0.1 nm of E2 (Fig. 3B). The transactivation abilities of ERα WT and SYN‐opt proteins were then analysed in the presence or absence of different ligands, including E2 (10 nm), the Selective oestrogen Modulator (SERM) 4‐hydroxytamoxifen (OHT, 1 μm) and the Selective Receptor Degrader (SERD) ICI 182–780 (ICI, 100 nm). The ERE‐driven luciferase reporter gene used was the C3‐Luc because of its high sensitivity to the agonist activity of OHT. Interestingly, ERα SYN‐opt exhibited a lower ligand‐independent activity than ERα WT on the C3‐Luc reporter gene (Fig. 3C). This activity was then induced nearly three‐ or five‐fold in the presence of OHT or E2, respectively, whereas it was completely inhibited by ICI. Besides binding directly to ERE‐driven genes, ERα can also regulate gene expression indirectly through protein–protein interactions with other transcriptional regulators such as activating protein 1 (AP1) and specific protein 1 (SP1). OHT and ICI were previously described as potent agonists on this ERα tethering pathway [39, 40, 41]. We, therefore, compared the transactivation activity of ERα WT and SYN‐opt proteins on AP1‐Luc and SP1‐Luc reporter genes after treatment of cells with E2, OHT or ICI (Fig. 3C). As expected, ERα WT was capable to transactivate both reporter genes after an OHT or ICI treatment and E2 treatment was inefficient on the AP1‐Luc. Surprisingly, ERα SYN‐opt protein was unable to mediate a transcriptional activation of both reporter genes, regardless of the ligand used. Slight residual activity was observed with ICI treatment only. Thus, synonymous mutations abolish ERα transactivation on AP1‐ and SP1‐driven reporter genes.

In conclusion, these results show that adapting the codon usage frequency of ERα by synonymous mutations impacts the ability of ERα to transactivate both classical (direct ERE binding) and non‐classical (tethering) reporter target genes.

### 3.3 ERα SYN‐opt protein enhances the transcriptional activity mediated by AF1

**Fig. 4:**
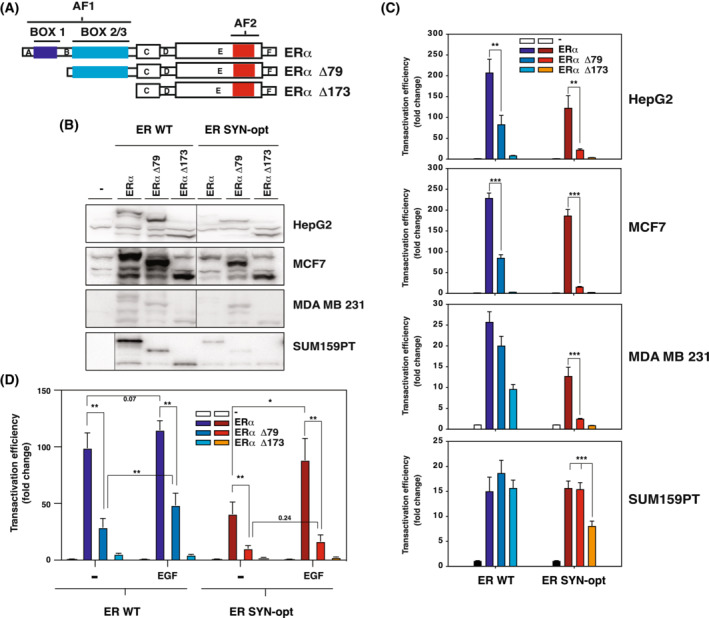
ERα SYN‐opt protein displays an enhanced AF1 activity. (A) Scheme of the sequences of ERα and the two N‐terminal truncated forms, ERα Δ79 and ERα Δ173, used in the study. (B) HepG2, MCF7, MDA‐MB‐231 and SUM159PT cells were transfected with pCR3.1, pCR ERα WT, pCR ERα WT Δ79, pCR ERα WT Δ173, pCR ERα ERα SYN‐opt, pCR ERα SYN‐opt Δ79 or pCR ERα SYN‐opt Δ173 expression vectors. And 48 h post‐transfection, expression of the different ERα forms was evaluated by western blot. A vertical line separates samples that were on the same western blot but not contiguous. (C) These same cell lines were transfected with the expression vectors previously described together with the C3‐Luc and CMV‐βgal reporter genes and then treated with 10 nm E2. Luciferase activity was normalized to β‐galactosidase activity. Data correspond to the mean values ± SEM of three (MCF7, MDA‐MB‐231 and SUM159PT) or six (HepG2) separate transfection experiments and are expressed as fold change from pCR3.1 control (**P‐value < 0.01 and ***P‐value < 0.001, Student's *t*‐test). (D) HepG2 cells were transfected with the expression vectors previously described together with the C3‐Luc and CMV‐βgal reporter genes, and then treated with 10 nm E2 in the presence or the absence of 50 ng·mL^−1^ of epidermal growth factor (EGF). Luciferase activity was normalized to β‐galactosidase activity. Data correspond to the mean values ± SEM of three separate transfection experiments and are expressed as fold change from pCR3.1 control (*P‐value < 0.05 and **P‐value < 0.01, Student's *t*‐test).

As previously mentioned, the transcriptional activity of ERα relies on two transactivation functions, AF1 and AF2 located in the N‐ and C‐terminal domains of the protein respectively. Specifically active in differentiated cells [11, 12], the AF1 has several different structural and functional units referred to as boxes 1–3 (Fig. 4A). We, therefore, evaluated the respective contribution exerted by both AF2 and AF1 boxes towards the transcriptional activity of ERα SYN‐opt protein. For that purpose, we generated for each ERα WT and SYN‐opt proteins, two N‐terminal deleted forms, ERα ∆79 (deletion of AF1 box 1) and ERα ∆173 (additional deletion of AF1 boxes 2/3). We then compared the transcriptional activity of these deleted forms with that of the full‐length ERα in different cell lines with various differentiation stage and AF1 permissiveness [11, 12, 35]. A similar activity measured for full‐length ERα and ERα ∆79 indicates the absence of an AF1 box 1 activity when a strict AF2‐mediated transcriptional activity is revealed by the same activity for full‐length ERα and ERα ∆173. The expression of the different forms was first controlled by Western blotting (Fig. 4B). The full‐length and deleted forms of ERα SYN‐opt were generally slightly less expressed that those of ERα WT. In the strict AF1‐permissive cell lines HepG2 and MCF7, ERα WT activity is mediated by both AF1 box 1 and box 2/3 as expected (Fig. 4C). Interestingly, ERα SYN‐opt favours the contribution of AF1 box 1 in these cells, since the difference of activity between the full‐length and ∆79 form is much higher in the context of the SYN‐opt receptor as compared to the WT ERα. In MDA‐MB‐231 cells, ERα WT activity does not require the AF1 box 1 activity, while ERα SYN‐opt activity remains primarily driven by this subdomain (Fig. 4C). Finally, whereas the transcriptional activity of ERα WT relies strictly on the AF2 function in the less‐differentiated cell line SUM159PT, ERα SYN‐opt additionally exhibits an AF1 boxes 2/3 activity, but AF1 box 1 activity remains quenched in these cells.

AF1 activity is also known to be regulated by growth factors such as the epithelial growth factor (EGF), through the phosphorylation of the serine residue at position 118 of ERα by the mitogen‐activated protein kinase MAPK [42]. We, therefore, evaluated the impact of EGF treatment on the transcriptional activity of ERα WT and SYN‐opt proteins. Results show that ERα SYN‐opt is slightly more sensitive to EGF stimulation than ERα WT (Fig. 4D). Notably, the effect of EGF appears to affect more the AF1 box 1 activity in ERα SYN‐opt protein when it depends more on box 2/3 activity in ERα WT protein.

Altogether, these results demonstrate that optimizing the codon usage frequency of ERα by synonymous mutations improves the transcriptional activity of AF1 function.

### 3.4 ERα SYN‐opt protein improves tamoxifen‐mediated repression in the presence of NCoR1 corepressor

**Fig. 5:**
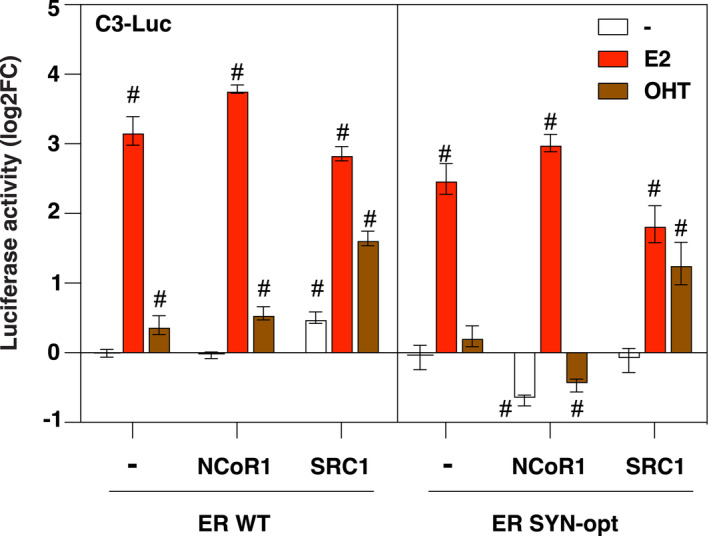
ERα SYN‐opt protein improves tamoxifen‐mediated repression in the presence of corepressor. MCF7 cells were transfected with pCR ERα WT or pCR ERα ERα SYN‐opt in the presence or the absence of pCR3.1, pCR‐SRC1 (Steroid receptor coactivator 1) or pCR‐NCoR1 (Nuclear receptor corepressor 1) expression vectors together with the C3‐Luc and CMV‐βgal reporter genes and then treated or not for 24 h with E2 (10 nm), OHT (1 μm), ICI (100 nm) or vehicle. Luciferase activity was normalized to β‐galactosidase activity. Data correspond to the mean values ± SEM from three separate transfection experiments and are expressed in log2 fold change (log2FC) from vehicle‐treated pCR3.1 control. Columns with # superscript differ significantly from non‐treated pCR3.1 control (*P* < 0.05, Student's *t*‐test).

SERMs such as OHT act as either agonists or antagonists of ERα functions through the recruitment of coactivators or corepressors, respectively, in a tissue‐, cell‐ and promoter‐specific manner [43, 44]. In an attempt to study the consequences of ERα codon optimization on the SERM activity of OHT, MCF7 was transiently transfected with either ERα WT or ERα SYN‐opt expression vectors in the presence or absence of expression vectors for SRC1 (steroid receptor coactivator‐1) or NCoR1 (nuclear receptor corepressor‐1), and the activity of the reporter gene C3‐Luc was then measured after treatment or not with E2 or OHT. We did not include ICI in these analyses since this molecule has no SERM activity on ERE‐driven genes. To better visualize the expected repressive activities, the results of these assays, shown in Fig. 5, were expressed in log2 fold change (FC) relative to the basal transcriptional activity of the reporter gene. Overexpression of SRC1 similarly enhanced the agonist activity of OHT on ERα WT and SYN‐opt proteins. In contrast, the overexpression of NCoR1 allowed only the ERα SYN‐opt protein to behave as a transcriptional repressor in the presence of OHT, but also, interestingly, in the absence of ligand. These results indicate that optimizing the codon usage frequency of ERα by synonymous mutations enhances the ability of ERα to repress transcription in the presence of OHT, presumably through improved NCoR1 corepressor recruitment.

### 3.5 E2 anti‐apoptotic effect is abolished in MCF7 expressing ERα SYN‐opt protein, resulting in a weaker oestrogen‐induced proliferation

**Fig. 6:**
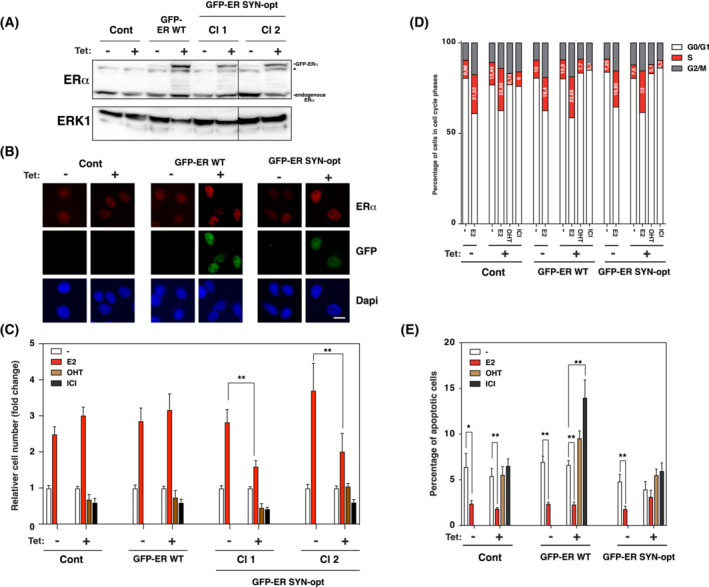
ERα SYN‐opt impairs oestrogen‐induced cell proliferation of MCF7 cells by abolishing the anti‐apoptotic effect of E2. (A) Control, GFP‐ERα WT and GFP‐ERα SYN‐opt MCF7 subclones were treated or not with tetracycline. And 48 h later, the expression of the different ERα proteins was analysed by western blot using ERα antibody. ERK1 expression was used as loading control. GFP‐ERα and endogenous ERα proteins are indicated. *: non‐specific band. A vertical line separates samples that were on the same western blot but not contiguous. The experiment was performed once. (B) GFP and immunofluorescent detection of ERα, 48 h after treatment or not with tetracycline of the control, GFP‐ERα WT and GFP‐ERα SYN‐opt MCF7 subclones. Nuclei were stained with DAPI. The experiment was repeated twice with similar results. Scale bar = 10 μm. (C) MCF7 subclones were treated or not with tetracycline together with E2 (10 nm), OHT (1 μm), ICI (100 nm) or vehicle for 6 days. The cells were counted manually upon trypsinization. Data correspond to the mean values ± SEM from four separate experiments and are expressed as fold change from cells treated with vehicle. E2 treatment values with ** superscript differ significantly between tetracycline‐treated and non‐treated value (*P* < 0.01, Student's *t*‐test). (D) MCF7 subclones were treated or not for 48 h with tetracycline together with E2 (10 nm), OHT (1 μm), ICI (100 nm) or vehicle during the last 24 h. Flow cytometry experiments were performed to determine the percentage of cells in each cell cycle phase. Data correspond to the mean values from three separate experiments. (E) MCF7 subclones were treated or not for 48 h with tetracycline together with E2 (10 nm), OHT (1 μm), ICI (100 nm) or vehicle during the last 24 h. Percentage of apoptotic cells was determined by terminal deoxynucleotidyl transferase dUTP nick end labelling (TUNEL). Data correspond to the mean values from triplicate experiments ± SEM (*P‐value < 0.05 and **P < 0.01, Student's *t*‐test).

To investigate the impact of ERα SYN‐opt protein on hormone‐dependent breast cancer cells, we generated MCF7 subclones with a tetracycline‐inducible vector system to stably expressed ERα SYN‐opt protein. We selected two clones, MCF7 ERα SYN‐opt clones 1 and 2. MCF7 subclones expressing either the empty expression vector (MCF7 control) or the expression vector encoding ERα WT (MCF7 ERα WT) were also established in parallel and used as controls. ERα WT and SYN‐opt proteins were fused to the GFP tag in their N‐terminal part in order to discriminate artificially expressed forms of ERα from endogenous ERα in MCF7 cells. Tetracycline‐induced expression of GFP‐ERα WT and SYN‐opt proteins was first controlled by western blot and immunofluorescence approaches (Fig. 6A,B). Their expression was predominantly localized in the nucleus, like endogenous ERα, with an approximately 1.5‐fold higher level for GFP‐ERα WT than for GFP‐ERα SYN‐opt protein. This difference is very likely due to changes in translational efficiency since GFP‐ERα WT and SYN‐opt mRNAs have similar expression levels and identical lengths with no detected splicing events in MCF7 subclones (Fig. S3). Interestingly, while the nuclear expression level of GFP‐ERα WT varies greatly from one cell to another in accordance with what is observed with the endogenous ERα, GFP‐ERα SYN‐opt shows a very similar expression between the different cells (Fig. S3). Furthermore, nuclear expression of GFP‐ERα SYN‐opt remains stable in mitotic cells identified by histone 3 serine 10 (H3S10) phosphorylation staining whereas that of GFP‐ERα WT drops sharply. We then assessed the impact of GFP‐tagged protein expression on cell proliferation. For that purpose, MCF7 control, GFP‐ERα WT and GFP‐ERα SYN‐opt cells were cultivated during 6 days in the presence or absence of tetracycline and under oestrogen or antioestrogen treatment, before counting the resulting cell population. As expected, E2 induced a threefold increase in cell numbers after 6 days of treatment when OHT and ICI slightly inhibited basal cell proliferation. For control and GFP‐ERα WT MCF7 cells, tetracycline treatment had no major effect. In contrast, cell counting of GFP‐ERα SYN‐opt MCF7 subclones clearly showed a twofold reduction in cell number following tetracycline treatment, indicating that GFP‐ERα SYN‐opt expression disrupts E2‐induced cell proliferation (Fig. 6C). Because similar results were obtained with GFP‐ERα SYN‐opt subclones 1 and 2 on cell proliferation, subsequent experiments were performed on subclone 1 only. Cell number results from the balance between cell cycle entry and apoptosis. These two parameters were, therefore, measured in order to determine the mechanism responsible for the limited E2‐induced proliferation of breast cancer cells following GFP‐ERα SYN‐opt expression. First, the relative proportion of cells in each phase of the cell cycle was analysed by flow cytometry (Fig. 6D). Results show a similar dynamic in the different cell cycle phases whatever the MCF7 subclones used and tetracycline treatment status. Hence, as expected, as compared to untreated cells, E2 enhanced twofold the percentage of cells in S phase and OHT and ICI treatments slightly reduced this cell sub‐population. The expression of GFP‐ERα WT or GFP‐ERαSYN‐opt proteins had no impact on the proportion of MCF7 cells in the different phases of the cell cycle. The percentage of apoptotic cells was then determined by TUNEL staining in each condition. In control and GFP‐ERα WT MCF7 cells, E2 treatment leads to a decrease in the number of apoptotic cells, whereas OHT and ICI treatments either had no strong effect or at most weakly stimulated apoptosis (Fig. 6E). Interestingly, when GFP‐ERα SYN‐opt protein was expressed in MCF7 cells, almost no reduction in the number of apoptotic cells was observed in presence of E2. Therefore, E2 loses its anti‐apoptotic properties in the presence of ERα SYN‐opt protein.

In conclusion, these results demonstrate that the low E2‐induced proliferation of MCF7 cells expressing of ERα SYN‐opt protein is not due to a defect in cell cycle entry but rather to an increased apoptosis.

### 3.6 Expression of ERα SYN‐opt protein in MCF7 cells alters MAPK and AKT signalling pathway

**Fig. 7:**
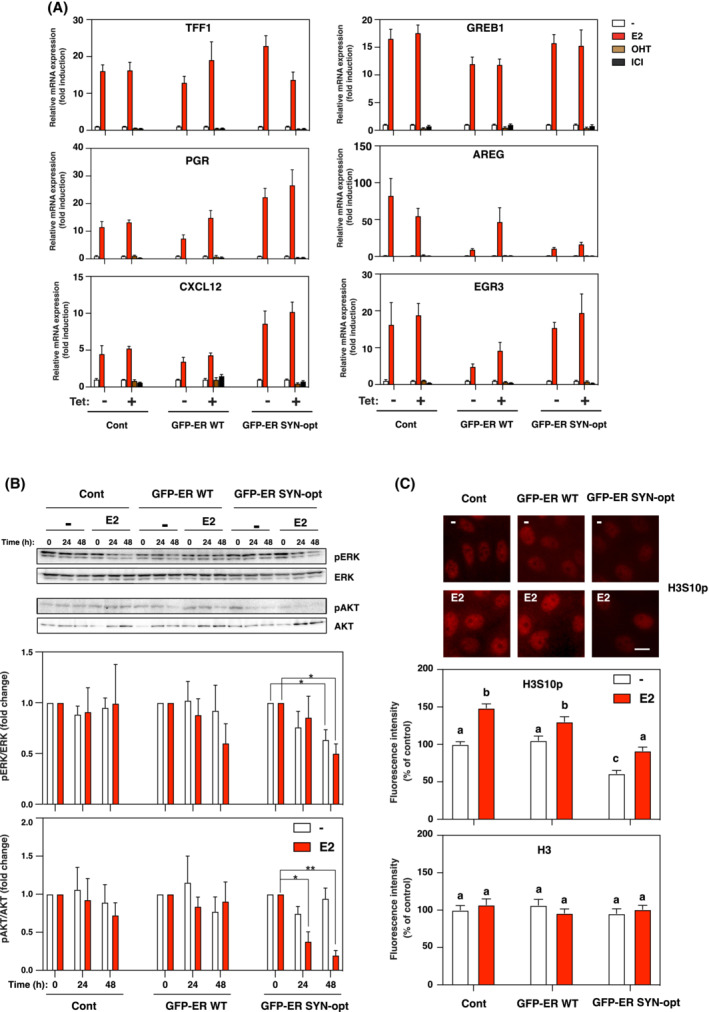
Expression of ERα SYN‐opt in MCF7 impacts MAPK and AKT signalling pathways rather than the E2‐target gene expression. (A) Control, GFP‐ERα WT and GFP‐ERα SYN‐opt MCF7 subclones were treated or not for 48 h with tetracycline together with E2 (10 nm), OHT (1 μm), ICI (100 nm) or vehicle during the last 24 h. The expression of the E2‐target genes, TFF1, PGR, CXCL12, GREB1, AREG and EGR3, were quantified using RT‐qPCR. Expression of E2‐target genes was normalized to TBP expression. Data correspond to the mean values ± SEM from three separate experiments and are expressed as fold change from vehicle‐treated control. (B) MCF7 subclones were treated with tetracycline in the presence or absence of 10 nm E2. Cells were harvested at 0, 24 and 48 h of treatment, and western blots were then performed to analyse phospho‐ERK (pERK), ERK, phospho‐AKT (pAKT) and AKT expression. Histograms represent the mean ± SEM of pERK/ERK or pAKT/AKT ratios from four separate experiments. Results were expressed as fold change from non‐treated control of each MCF7 subclones (*P‐value < 0.05 and **P‐value < 0.01, Student's *t*‐test). (C) MCF7 subclones were treated for 48 h with tetracycline in the presence or absence of 10 nm E2 during the last 24 h. On the top, immunofluorescence images of phosphorylation of histone 3 at serine 10 (H3S10p). Scale bar = 10 μm. On the bottom, densitometry quantification of the immunofluorescence images of H3S10p and total H3 (H3) expressed as percentage of the intensity measured in untreated (−E2) control cells. Values represent the mean ± SEM. Columns with the superscripts a, b and c differ significantly between each superscript (*n* = 20, *P*‐value < 0.05, Student's *t*‐test).

In MCF7 cells, ERα is known to exhibit both genomic and non‐genomic effects [14]. We, therefore, undertook the study of these two modes of action of ERα in the different MCF7 subclones. First, the genomic activities of the GFP‐ERα forms were investigated by studying the consequences of their expression on the activity of endogenous E2‐target genes. To do so, cells were treated or not for 48 h with tetracycline in the presence or absence of E2, OHT or ICI, and the expression of six main E2‐regulated genes, *TFF1*, *PGR*, *CXCL12*, *GREB1*, *AREG* and *EGR3*, was assessed by RT‐qPCR (Fig. 7A). Results show some variations in gene expression between the different subclones but which were not attributed to tetracycline‐induced expression of the proteins of interest. Indeed, while the expression levels of the studied genes were induced by E2 and slightly repressed in the presence of OHT and ICI, the expression of either GFP‐ERα WT or GFP‐ERα SYN‐opt proteins had clearly no impact on these regulations. These data suggest that ERα WT and SYN‐opt proteins expression in MCF‐7 cells do not impact endogenous ERα activity on endogenous E2‐responsive genes.

Non‐genomic activities of GFP‐ERα forms were then investigated by examining the phosphorylation status of the extracellular signal‐regulated kinases (ERK) and AKT by western blot in control, GFP‐ERα WT and GFP‐ERα SYN‐opt MCF7 subclones after 24 and 48 h treatment with tetracycline alone or with E2. No significant alteration of ERK nor AKT phosphorylations was observed in control and GFP‐ERα WT MCF7 cells following tetracycline and E2 treatments. On the contrary, in GFP‐ERα SYN‐opt MCF7 subclone, the addition of tetracycline resulted in the decreased phosphorylations of ERK or AKT in an E2‐independent or E2‐dependent manner respectively. Accordingly, in contrast to ERα WT, the ERα SYN‐opt represses MAPK and AKT signalling pathway (Fig. 7B). Although non‐genomic activity of ERα is primarily characterized by processes affecting components of signal transduction pathways, the subsequent cellular response may ultimately resume to genes expression regulations through the phosphorylation of transcription factors, coregulators or structural components of chromatin. Notably, histone H3 phosphorylation at serine 10 (H3S10p) is one of the main nucleosome responses to mitogen or growth factor signalling pathways through the activation of the mitogen and stress‐activated kinase 1 (MSK1) [45]. Since steroid hormones including progestin and oestrogen have been shown to induce this signalling pathway [46, 47], we investigated histone H3 phosphorylation status at serine 10 by immunofluorescence in the different MCF7 subclones treated or not 24 h with E2. Results showed that E2 increased H3S10 phosphorylation in all three MCF7 subclones, nevertheless with a lower basal level of H3S10p in GFP‐ERα SYN‐opt MCF7 cells than in control and GFP‐ERα WT MCF7 cells (Fig. 7C). Taken together, these results lead to the conclusion that the repression of MAPK signalling pathway as occurs in GFP‐ERα SYN‐opt MCF7 cells converges at the chromatin level through repression of H3S10 phosphorylation.

### 3.7 ERα SYN‐opt interacts more with corepressors and less with kinases than ERα WT in MCF7 cells

**Fig. 8:**
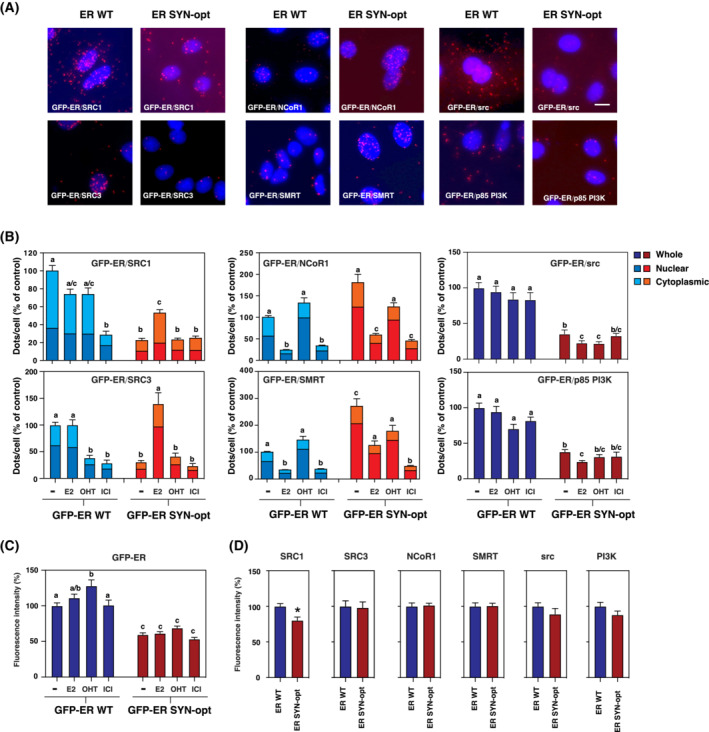
ERα SYN‐opt interacts more with corepressors and less with kinases than ERα WT in MCF7 cells. GFP‐ERα WT and GFP‐ERα SYN‐opt MCF7 subclones were treated 48 h with tetracycline. At the end of treatment, cells were stimulated with E2 (10 nm), OHT (1 μm), ICI (100 nm) or vehicle during 60 min for the study of ERα/cofactor interactions and during 10 min for the study of ERα/kinase interactions. GFP‐ERα WT or GFP‐ERα SYN‐opt complexes with coactivators (SRC1 and SRC3), corepressors (NCoR1 and SMRT) and kinases (Src and PI3K) were detected by PLA. (A) Representative pictures of the experiments are shown with DAPI‐stained nuclei. Scale bar = 10 μm. (B) Quantification of the number of dots/cell and dots/nucleus was performed using imagej software and was then expressed as a percentage of the number of dots/cell measured in vehicle‐treated GFP‐ERα WT MCF7 subclone. The respective proportion of complexes in the nucleus (N) and in the cytoplasm (C) is shown. Values represent the mean ± SEM. Columns with the superscripts a, b and c differ significantly between each superscript (*n* = 20, *P*‐value < 0.001, Student's *t*‐test). (C) Quantification of the expression level of GFP‐ERα WT and GFP‐ERα SYN‐opt through GFP fluorescence, 60 min after treatment with ligands. Values represent the mean ± SEM. Columns with the superscripts a, b and c differ significantly between each superscript (*n* = 20, *P*‐value < 0.001, Student's *t*‐test). (D) Quantification of the expression level of SRC1, SRC3, NCoR1, SMRT, Src and PI3K in GFP‐ERα WT and GFP‐ERα SYN‐opt MCF7 subclones after immunofluorescence detection. Values represent the mean ± SEM (*n* = 10, *P‐value < 0.05, Student's *t*‐test).

Because ERα SYN‐opt protein was found to impact E2 signalling pathway in MCF7 cells, we extended our study to analyse the interaction of ERα with some of its main partners such as (a) members of the steroid coactivator P160 family, SRC1 (steroid receptor coactivator 1) and SRC3 (steroid receptor coactivator 3; AIB1); (b) the corepressors NCoR1 (nuclear receptor corepressor 1) and SMRT (NcoR2; nuclear receptor corepressor 2); and (c) the kinases Src and PI3K. After treating GFP‐ERα WT and GFP‐ERα SYN‐opt MCF7 subclones with the different ligands E2, OHT or ICI, protein interactions were measured by proximity ligation assay (PLA) using antibodies targeting either the GFP‐ERα protein through the GFP tag or Erα's partners. Confirming our previous western blot analyses, quantification of the GFP fluorescence intensity shows that GFP‐ERα SYN‐opt expression is almost 1.5‐fold lower than that of GFP‐ERα WT and that a short‐term treatment with either ligand has little impact on the protein expression level (Fig. 8C). As expected, the interaction profiles of GFP‐ERα WT with the tested coactivators, corepressors and kinases were similar to those previously described with the endogenous receptor [17]. For instance, as previously reported, the interaction of ERα with P160 family coactivators is not further enhanced by E2 but strongly inhibited in the presence of ICI. Additionally, the interaction of ERα with NCoR1 and SMRT corepressors was almost abolished by a treatment with E2 as well as ICI but persistent after cells exposure to OHT. Finally, the ERα/kinase complex formation was not or only partially affected by ligands [17]. The similarity of GFP‐ERα WT interaction profiles with those of the endogenous receptor indicates that the fusion of GFP to the N‐terminal domain of ERα does not affect the interactions of the receptor with the tested partners. Interestingly, notable differences were observed when assessing the interaction of the GFP‐ERαSYN‐opt with its partners (Fig. 8B). First, as compared to the GFP‐ERα WT, the GFP‐ERαSYN‐opt interacted less with SCR1 and SRC3 coactivators when in a basal state. Further changes in these interactions with coactivators occurred only when cells were treated with E2. The interactions of GFP‐ERα WT and GFP‐ERαSYN‐opt with NCoR1 and SMRT corepressors and Src and PI3K kinases were similarly affected by the three tested ligands. However, as compared to the GFP‐ERα WT, we detected more ERα/corepressor complexes and less ERα/kinase complexes in the case of the GFP‐ERαSYN‐opt protein. These differences in interaction intensity cannot fully be attributed to differences in the expression level of the corresponding proteins involved in the interaction (Fig. 8C,D). Compared with a same amount of ERα protein, the interaction of GFP‐ERα SYN‐opt with the two corepressors increases further and its interaction with Src and PI3K kinases still remains twofold weaker than that observed with GFP‐ERα WT. Therefore, taken together, these results indicate that optimizing the codon usage frequency of ERα by synonymous mutations affects how ERα protein interacts with its partners, specifically favouring interaction with NCoR1 and SMRT corepressors over interactions with Src and PI3K kinases.

### 3.8 Limited digestions of ERα WT and ERα SYN‐opt proteins suggest conformational differences

**Fig. 9:**
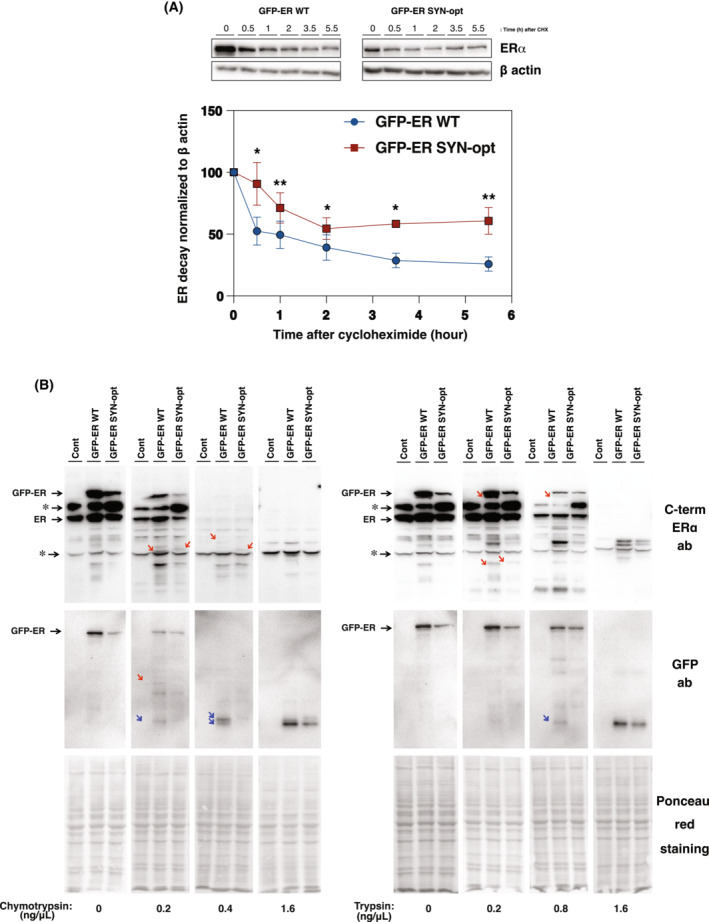
Limited digestions of ERα WT and ERα SYN‐opt proteins suggest conformational differences. (A) Time course of ERα WT and ERα SYN‐opt degradation. GFP‐ERα WT and GFP‐ERα SYN‐opt MCF7 subclones were treated with tetracycline for 48 h. Cells were then harvested at different times (0, 0.5, 1, 2, 3.5 and 5.5 h) after treatment with 20 μg·mL^−1^ cycloheximide. ERα WT and ERα SYN‐opt protein expression was analysed by western blot. β actin expression was used as a control. Histograms represent the mean ± SEM of ERα WT and ERα SYN‐opt protein expressions normalized to β actin expression from four separate experiments. Results were expressed as a percentage of ERα expression at time 0 h. A Student's *t*‐test was performed at each time point between ERα WT and ERα SYN‐opt expression (*P‐value < 0.05 and **P‐value < 0.01). (B) Limited digestions of ERα WT and ERα SYN‐opt proteins. Control, GFP‐ERα WT and GFP‐ERα SYN‐opt MCF7 subclones were treated for 48 h with tetracycline. Limited digestions of cell lysates by increasing concentrations of trypsin or chymotrypsin (0.2–1.6 ng·μL^−1^) were analysed by western blot using antibodies raised against ERα C‐terminal region and GFP. Ponceau red staining was used to validate the homogeneity of protein amounts between each sample. * indicates non‐specific band. Red arrows indicate several digested products of different weight between GFP‐ERα WT and GFP‐ERα SYN‐opt MCF7 cell lysates. Blue arrows indicate several digested products with different degradation kinetic between GFP‐ERα WT and GFP‐ERα SYN‐opt MCF7 cell lysates. The experiment was repeated twice with similar results.

Changes in the functional properties of ERα SYN‐opt compared with ERα WT, both in transcriptional activity and partner interactions, strongly suggest differences in the conformation of the two proteins. In this continuity, a potential modification of its conformation was further investigated by first studying protein degradation kinetics and then by performing limited digestions. Protein degradation kinetic was analysed by western blot after blocking the protein synthesis in GFP‐ERα WT and GFP‐ERα SYN‐opt MCF7 subclones with cycloheximide (Fig. 9A). Results shows a significant increase in the stability of the GFP‐ERα SYN‐opt as compared to the GFP‐ERα WT protein, suggesting possible different protein conformations. Importantly, these results additionally indicate that the observed lower amounts of ERα SYN‐opt protein produced in the cells (Figs 2, 3, 4, 6 and 8), as compared to GFP‐ERα WT, are more related to a lower efficiency of mRNA translation than to an increased degradation of the protein. Next, we performed limited digestion experiments to assess for putative differing conformation of ERα WT and SYN‐opt proteins more directly. For that purpose, increasing amounts of chymotrypsin or trypsin were incubated with the same quantity of cell lysate from GFP‐ERα WT and GFP‐ERα SYN‐opt MCF7 subclones. The resulting protein extracts from of these limited digestions were then resolved by Western blotting using C‐terminal ERα and GFP antibodies. As shown within Fig. 9B, the digestions of GFP‐ERα WT and GFP‐ERα SYN‐opt proteins exhibited some differences in terms of digestion kinetics and weight of digested peptides. Indeed, the degradation kinetics of GFP‐ERα SYN‐opt by both digesting enzymes appeared indeed slower than that of GFP‐ERα WT, in agreement with the conclusion rose from the previous experiment (Fig. 9A). This is particularly visible with the anti‐GFP antibody which targets the N‐terminal part of the GFP‐fused ERα proteins. Additionally, several digested products of different weights were detected when comparing GFP‐ERα WT to GFP‐ERα SYN‐opt MCF7 cell lysates (indicated by red arrows on Fig. 9B). Altogether, these results obtained by following the degradation kinetics of WT and SYN‐opt ERα proteins and their profiles of limited digestions strongly suggest that these proteins have different conformations.

## 4 Discussion

The proliferation of ERα‐positive breast cancer cells can be inhibited by using antioestrogens. Unfortunately, endocrine resistance often occurs, resulting in the oestrogen‐independent development of cancerous cells. Many works aimed to understand the mechanisms leading to this hormonal escape and identified notably the role played by the imbalance of coregulators and growth factor signalling pathways. It has indeed been shown that the relative expressions of P160 family coactivators and NCoR1 and SMRT corepressors modulate the activity of the antioestrogen tamoxifen toward an ERα agonism or antagonism. This, for instance, explains why tamoxifen exerts an agonist effect in the uterus where the level of coactivators is increased, as compared to breast cancer cells where tamoxifen acts as an ERα antagonist [44]. The overexpression of coactivators such as SRC1 and the repression of corepressors such as NCoR1 in breast cancer cells have then been associated with tamoxifen resistance [48]. As intracellular kinases are able to activate ERα by phosphorylation, the overactivation of components of the MAPK or PI3K pathways plays a role in hormonal escape, by promoting the ligand‐independent activation of ERα. This dysregulated activation results from mutations that are frequent in ERα‐positive breast cancers, or the overexpression of growth factor receptors that activate such pathways, such as the EGF receptor (EGFR), which has been linked to endocrine resistance as well [15].

In healthy breast tissue, ERα is expressed in a small number of luminal cells, a subset of differentiated epithelial cells that are not proliferating, as assessed by a lack of co‐staining with the proliferating marker Ki67 [49, 50]. In this context, ERα exerts its oestrogen‐induced mitogen activity by a paracrine mechanism, favouring the proliferation of surrounding ERα‐negative cells only [51]. In ERα‐positive breast cancers, however, an increased proportion of ERα‐expressing cells appears to be proliferating, due to a shift from a paracrine to an autocrine regulation of proliferation by ERα [49, 52]. This change from quiescent to proliferating ERα‐expressing cells during tumour transformation results in different modifications in cell activity that can lead to changes in functional properties of ERα explaining endocrine resistance.

As previous works highlighted tRNA pool variations between differentiated and proliferating cells [24], we hypothesized that this could occur in proliferative ERα‐positive breast cancer cells compared to non‐cancerous ERα‐expressing cells that remain quiescent. These modifications of tRNA pools could then alter the translation speed of ERα, resulting in an alteration of its co‐translational folding. This would ultimately lead to a modification of its conformation, explaining the dysregulation of ERα functions in breast cancer cells, participating to hormonal escape. This tRNA pool variation was correlated to a different codon usage bias in genes specifically expressed in differentiated or proliferating cells, suggesting an adequacy between codon usage and tRNA pool to enable the correct production of proteins in specific cell states [24].

We first evidenced that ERα encoding mRNA contains codons whose usage frequency is associated with differentiation programme. We, therefore, reasoned that ERα production and/or conformation would likely differ between dedifferentiated and proliferating cells. We stressed this hypothesis by generating a synonymous version of ERα by adapting the frequency of its codon usage to that observed in genes specifically expressed in proliferative cells, allowing us to investigate if this sequence optimization affects ERα functional properties in cancerous cells. Because this adaptation results in significant changes in the coding nucleotide sequence that can impact transcriptional and translation efficiency, we first verified that the synonymous version of ERα (ERα SYN‐opt) was correctly produced both qualitatively and quantitatively. While the expression levels of both mRNAs were identical, we observed a slightly attenuated production of the ERα SYN‐opt protein as compared to ERα WT, probably due to a reduced translational efficiency. Nevertheless, ERα SYN‐opt remains abundant and functional. Interestingly, several of our results clearly demonstrate that codon adaptation to expression in proliferating cell indeed restores functional properties of ERα normally observed in differentiated cells: First, in MCF7, endogenous ERα nuclear expression is high in quiescent cells and is downregulated during cell cycle [53], resulting in heterogeneous expression of ERα in asynchronous cells. But the adaptation of ERα codons to those used in proliferating cells, as occurs in ERα SYN‐opt, led to the homogenization of ERα expression in MCF7 cells with no downregulation in mitotic cells. Second, in differentiated cells, ERα exerts a genomic activity mainly driven by its AF1 function, especially its box 1 structural unit [11, 12]. But optimizing the codon usage of ERα for proliferating cells improved the transcriptional activity mediated by AF1 box 1 even in dedifferentiated cells. Third, hormone‐dependent breast cancers are usually more differentiated and less invasive than the other types of breast cancers and generally respond well to endocrine therapy [7]. In particular, ERα transcriptional activity in breast cancer cells responding to endocrine therapy is repressed by the 4‐hydroxytamoxifen through corepressor recruitment by ERα [43, 54]. As mentioned previously, one mechanism of endocrine resistance is an imbalance in ERα recruitment between P160 family coactivators and NCoR1 and SMRT corepressors in favour of coactivators. We clearly show that the ability of OHT to repress transcription was increased after ERα codon adaptation, through improved NCoR1 and SMRT corepressor recruitment, which was further validated by PLA in MCF7 cells. Finally, non‐genomic activity of ERα, which leads to the phosphorylation of intracellular kinases involved in MAPK or AKT signalling pathways, is mediated by the formation of complexes between ERα and kinases such as Src or PI3K. These complexes are increased in aggressive breast cancer cells, playing a role in endocrine resistance [17, 55]. We demonstrate that codon optimization of ERα reduces the interaction of ERα with Src and PI3K kinases and represses MAPK and AKT signalling pathways when the protein is stably expressed in MCF7. All these changes in ERα activity are likely related to changes directly affecting ERα protein, because only the codon sequence of ERα has been modified. Since protein degradation kinetics and limited digestions with trypsin or chymotrypsin showed differences between WT and codon optimized forms of ERα, the changes in ERα activity are likely based on differences in ERα conformation.

The impact of ERα SYN‐opt expression on MCF7 cell fate was also investigated in this study. While cells expressing this ERα SYN‐opt appear to enter S‐phase normally after oestrogenic stimulation, we found that these cells exhibited a lower E2‐induced proliferation rate than those expressing ERα WT, due to the abolition of the anti‐apoptotic effect of E2. There is no clear information regarding the apoptotic activity of ERα‐positive epithelial cells in healthy breast tissue, but it is noteworthy that ERα‐positive cells are few in number and represent about 10% of the total luminal epithelial cell population in human, which may suggest some sensitivity to apoptosis.

The observation of changes in ERα activity as a result of codon adaptation clearly reinforces the notion of distinct translation programmes. With specialized ribosomes [56], variation in the tRNA pool is one of the most effective ways to modify translation programmes [26, 29]. It is known that tRNA function or expression is affected under stress conditions [57], through cell cycle [24, 58], by cell‐fate [24, 59] or during tumorigenesis and cancer progression [25, 60]. We do not know whether the cell lines used in this study have different tRNA repertoires or not. However, using YAMAT‐seq method, Shigematsu et al. showed variations in the expression levels of mature tRNAs between three different subtypes of breast cancer cell lines, MCF7 (luminal A subtype), SK‐BR‐3 (HER2‐positive subtype) and BT‐20 (triple negative subtype) [61]. We recently demonstrated that the activation and nuclear accumulation of MRTFA, a master regulator of EMT, in the luminal breast cancer cell line MCF7 induces a basal‐like phenotype and remodels ERα functions by shifting its activity from nuclear genome regulation to extra‐nuclear non‐genomic signalling [17, 18]. Further studies are now needed to verify whether this change in ER activity is accompanied by a change in the tRNA repertoire.

## 5 Conclusion

The results obtained in this study attest that modifying the codons used to produce ERα can lead to functional modifications, resulting from alterations of its conformation. ERα SYN‐opt displays indeed a more repressive phenotype than ERα WT, meaning that adapting its codons in agreement with the frequencies observed in genes specifically expressed in proliferating cells enhanced the phenotype of ERα in differentiated epithelial cells. These data highlight a translational regulation of ERα conformation linked to its nucleotide sequence and cell state, likely due to the role played by codon usage in the translational process regulation. Such an observation could have important implications in understanding the biology of ERα from stem cells to differentiated cells and from healthy to cancerous tissues and will require further investigation.

## Conflict of interest

The authors declare no conflict of interest.

## Author contributions

GF conceived the project. LC, FPe, EJ, PLG, CT and GF conducted the experiments. TF‐C, RM, MM, FPa and DM contributed to the data analysis. LC, TF‐C, DM and GF wrote the manuscript with input from all authors.

### Peer review

The peer review history for this article is available at https://publons.com/publon/10.1002/1878‐0261.13399.

## Supporting information


**Fig. S1.** Codon usage optimization of ERα coding sequence by synonymous mutations.
**Fig. S2.** Regulatory elements, promoter/enhancer and secondary mRNA structure predictions from ERα WT and ERα SYN opt coding sequences.
**Fig. S3.** GFP‐ERα mRNA and protein expression in control, GFP‐ERα WT and GFP‐ERα SYN‐op MCF7 subclones.
**Table S1.** List of primers used to detect target genes by RT‐qPCR.

## Data Availability

Data supporting the results of this study are included in the supporting information or are available from the corresponding author (gilles.flouriot@univ-rennes1.fr) upon request.
