# Supplementary material for: Codon adaptation by synonymous mutations impacts the functional properties of the estrogen receptor‐alpha protein in breast cancer cells
Source: Mol Oncol. 2023 Mar 1;17(7):1302–23. doi: 10.1002/1878-0261.13399 (PMC10323882; doi:10.1002/1878-0261.13399)
Supplement: Supplementary file 1 — Fig. S1. Codon usage optimization of ERα coding sequence by synonymous mutations. Fig. S2. Regulatory elements, promoter/enhancer and secondary mRNA structure predictions from ERα WT and ERα SYN opt coding sequences. Fig. S3. GFP‐ERα mRNA and protein expression in control, GFP‐ERα WT and GFP‐ERα SYN‐op MCF7 subclones. Table S1. List of primers used to detect target genes by RT‐qPCR. [file MOL2-17-1302-s001.pdf]

A

		Codon usage frequency		
**Amino acid**	**Codon**	**proliferation**	**differentiation**	**ERα**
ala	gca	0,32	0,16	0,15
ala	gcc	0,3	0,46	0,51
ala	gcg	0,06	0,23	0,15
ala	gct	0,32	0,15	0,19
arg	aga	0,31	0,1	0,23
arg	agg	0,2	0,16	0,23
arg	cga	0,14	0,08	0,11
arg	cgc	0,11	0,33	0,26
arg	cgg	0,15	0,27	0,11
arg	cgt	0,09	0,06	0,06
asn	aac	0,42	0,75	0,76
asn	aat	0,58	0,25	0,24
asp	gac	0,42	0,75	0,67
asp	gat	0,58	0,25	0,33
cys	tgc	0,4	0,73	0,62
cys	tgt	0,6	0,27	0,38
gln	caa	0,36	0,17	0,16
gln	cag	0,63	0,83	0,84
glu	gaa	0,55	0,25	0,19
glu	gag	0,45	0,75	0,81
gly	gga	0,35	0,14	0,22
gly	ggc	0,25	0,49	0,41
gly	ggg	0,19	0,27	0,22
gly	ggt	0,2	0,1	0,15
his	cac	0,45	0,77	0,64
his	cat	0,55	0,23	0,36
ile	ata	0,23	0,08	0,11
ile	atc	0,34	0,71	0
ile	att	0,43	0,22	0,26
leu	cta	0,1	0,05	0,08
leu	ctc	0,14	0,26	0,22
leu	ctg	0,28	0,49	0,48
leu	ctt	0,17	0,08	0,04
leu	tta	0,13	0,04	0,01
leu	ttg	0,17	0,08	0,16
lys	aaa	0,5	0,26	0,34
lys	aag	0,5	0,74	0,66
met	atg	1	1	1
phe	ttc	0,43	0,69	0,73
phe	ttt	0,57	0,31	0,27
pro	cca	0,35	0,18	0,17
pro	ccc	0,24	0,39	0,46
pro	ccg	0,06	0,25	0,26
pro	cct	0,34	0,19	0,11
ser	agc	0,19	0,33	0,27
ser	agt	0,2	0,09	0,13
ser	tca	0,19	0,09	0,09
ser	tcc	0,16	0,23	0,2
ser	tcg	0,04	0,15	0,07
ser	tct	0,22	0,11	0,24
thr	aca	0,32	0,18	0,16
thr	acc	0,27	0,45	0,48
thr	acg	0,08	0,23	0,16
thr	act	0,33	0,14	0,20
trp	tgg	1	1	1
tyr	tac	0,41	0,74	0,70
tyr	tat	0,59	0,26	0,30
val	gta	0,18	0,08	0,04
val	gtc	0,16	0,3	0,27
val	gtg	0,4	0,52	0,65
val	gtt	0,27	0,1	0,04

B

```
WT            1 ATGACCATGACCCTCCACACCAAAGCATCTGGGATGGCCCTACTGCATCAGATCCAAGGG
SYN-opt       1 ATGACAATGACACTTCATACAAAGGCGTCCGGAATGGCCCTACTGCACCAGATTCAAGGA
                ***** ***** ** ** ** ** ** ** ** ************* ***** *****

WT           61 AACGAGCTGGAGCCCCTGAACCGTCCGCAGCTCAAGATCCCCCTGGAGCGGCCCCTGGGC
SYN-opt      61 AATGAACTGGAACCACTGAATCGTCCACAGCTTAAAATTCCACTGGAAAGACCACTGGGA
                ** ** ***** ** ***** ***** ***** ** ** ** *****  * ** *****

WT          121 GAGGTGTACCTGGACAGCAGCAAGCCCGCCGTGTACAACTACCCCGAGGGCGCCGCCTAC
SYN-opt     121 GAAGTGTATCTGGATAGTAGTAAACCAGCCGTGTATAATTATCCAGAAGGAGCCGCCTAT
                ** ***** ***** ** ** ** ** ******** ** ** ** ** ** ********

WT          181 GAGTTCAACGCCGCGGCCGCCGCCAACGCGCAGGTCTACGGTCAGACCGGCCTCCCCTAC
SYN-opt     181 GAATTTAATGCCGCTGCCGCCGCCAATGCTCAGGTTTATGGGCAGACAGGACTTCCATAT
                ** ** ** ***** *********** ** ***** ** ** ***** ** ** ** **

WT          241 GGCCCCGGGTCTGAGGCTGCGGCGTTCGGCTCCAACGGCCTGGGGGGTTTCCCCCCACTC
SYN-opt     241 GGACCAGGATCCGAAGCGGCTGCTTTTGGAAGTAATGGACTGGGAGGGTTTCCACCCCTT
                ** ** ** ** ** ** ** ** ** **    ** ** ***** ** ** ** ** **

WT          301 AACAGCGTGTCTCCGAGCCCGCTGATGCTACTGCACCCGCCGCCGCAGCTGTCGCCTTTC
SYN-opt     301 AATAGTGTGTCCCCAAGTCCACTGATGCTACTGCATCCACCACCACAGCTGAGTCCCTTT
                ** ** ***** ** ** ** ************** ** ** ** ******    ** **

WT          361 CTGCAGCCCCACGGCCAGCAGGTGCCCTACTACCTGGAGAACGAGCCCAGCGGCTACACG
SYN-opt     361 CTGCAGCCACATGGACAGCAGGTGCCATATTATCTGGAAAATGAACCAAGTGGATATACT
                ******** ** ** *********** ** ** ***** ** ** ** ** ** ** **

WT          421 GTGCGCGAGGCCGGCCCGCCGGCATTCTACAGGCCAAATTCAGATAATCGACGCCAGGGT
SYN-opt     421 GTGAGAGAAGCCGGACCACCAGCGTTTTATCGGCCCAACTCGGACAACCGCAGACAGGGG
                *** * ** ***** ** ** ** ** **  **** ** ** ** ** **  * *****

WT          481 GGCAGAGAAAGATTGGCCAGTACCAATGACAAGGGAAGTATGGCTATGGAATCTGCCAAG
SYN-opt     481 GGACGCGAGCGCCTAGCCTCTACAAACGATAAAGGGTCTATGGCGATGGAGTCCGCCAAA
                **  * **  *  * ***  *** ** ** ** **   ****** ***** ** *****

WT          541 GAGACTCGCTACTGTGCAGTGTGCAATGACTATGCTTCAGGCTACCATTATGGAGTCTGG
SYN-opt     541 GAAACGAGATATTGCGCGGTGTGTAACGATTACGCGTCGGGATATCACTACGGGGTTTGG
                ** **  * ** ** ** ***** ** ** ** ** ** ** ** ** ** ** ** ***

WT          601 TCCTGTGAGGGCTGCAAGGCCTTCTTCAAGAGAAGTATTCAAGGACATAACGACTATATG
SYN-opt     601 AGTTGCGAAGGATGTAAAGCCTTTTTTAAACGCTCTATCCAAGGGCACAATGATTACATG
                   ** ** ** ** ** ***** ** **     *   *** ***** ** ** ** ** ***

WT          661 TGTCCAGCCACCAACCAGTGCACCATTGATAAAAACAGGAGGAAGAGCTGCCAGGCCTGC
SYN-opt     661 TGCCCCGCCACAAATCAGTGTACAATCGACAAGAATCGGCGGAAAAGTTGTCAGGCCTGT
                ** ** ***** ** ***** ** ** ** ** **  ** **** ** ** ********

WT          721 CGGCTCCGCAAATGCTACGAAGTGGGAATGATGAAAGGTGGGATACGAAAAGACCGAAGA
SYN-opt     721 AGACTTAGAAAGTGTTATGAGGTGGGGATGATGAAGGGGGGAATACGCAAGGATCGCCGC
                 * **  * ** ** ** ** ***** ******** ** ** ***** ** ** **  *

WT          781 GGAGGGAGAATGTTGAAACACAAGCGCCAGAGAGATGATGGGGAGGGCAGGGGTGAAGTG
SYN-opt     781 GGGGGACGCATGCTAAAGCATAAAAGACAGCGCGACGACGGAGAAGGACGGGGGGAGGTG
                ** **  * *** * ** ** **  * *** * ** ** ** ** **  **** ** ***

WT          841 GGGTCTGCTGGAGACATGAGAGCTGCCAACCTTTGGCCAAGCCCGCTCATGATCAAACGC
SYN-opt     841 GGATCCGCGGGGGATATGCGCGCGGCCAATCTTTGGCCCAGTCCACTTATGATTAAGAGA
                ** ** ** ** ** *** * ** ***** ******** ** ** ** ***** **  *

WT          901 TCTAAGAAGAACAGCCTGGCCTTGTCCCTGACGGCCGACCAGATGGTCAGTGCCTTGTTG
SYN-opt     901 TCCAAAAAAAATAGTCTGGCCCTAAGTCTGACTGCCGATCAGATGGTTTCTGCCCTACTA
                ** ** ** ** ** ****** *    ***** ***** ********   **** *  *

WT          961 GATGCTGAGCCCCCCATACTCTATTCCGAGTATGATCCTACCAGACCCTTCAGTGAAGCT
SYN-opt     961 GACGCGGAACCACCAATACTTTACAGTGAATACGACCCCACACGCCCATTTTCTGAGGCG
                ** ** ** ** ** ***** **    ** ** ** ** **  * ** **   *** **

WT         1021 TCGATGATGGGCTTACTGACCAACCTGGCAGACAGGGAGCTGGTTCACATGATCAACTGG
SYN-opt    1021 AGTATGATGGGACTCCTGACAAATCTGGCGGATCGGGAACTGGTCCATATGATTAATTGG
                   ********  * ***** ** ***** **  **** ***** ** ***** ** ***

WT         1081 GCGAAGAGGGTGCCAGGCTTTGTGGATTTGACCCTCCATGATCAGGTCCACCTTCTAGAA
SYN-opt    1081 GCTAAACGGGTGCCCGGATTCGTGGACCTAACACTTCACGACCAGGTTCATCTTCTAGAG
                ** **  ******* ** ** *****  * ** ** ** ** ***** ** ********

WT         1141 TGTGCCTGGCTAGAGATCCTGATGATTGGTCTCGTCTGGCGCTCCATGGAGCACCCAGGG
SYN-opt    1141 TGCGCCTGGCTAGAAATTCTGATGATCGGGCTTGTTTGGAGAAGTATGGAACATCCCGGA
                ** *********** ** ******** ** ** ** *** *    ***** ** ** **

WT         1201 AAGCTACTGTTTGCTCCTAACTTGCTCTTGGACAGGAACCAGGGAAAATGTGTAGAGGGC
SYN-opt    1201 AAACTACTGTTCGCGCCCAATCTACTTCTAGATCGGAATCAGGGGAAGTGCGTCGAAGGA
                ** ******** ** ** **  * **  * **  **** ***** ** ** ** ** **

WT         1261 ATGGTGGAGATCTTCGACATGCTGCTGGCTACATCATCTCGGTTCCGCATGATGAATCTG
SYN-opt    1261 ATGGTGGAAATTTTTGATATGCTGCTGGCGACCTCGTCCAGATTTAGAATGATGAACCTG
                ******** ** ** ** *********** ** ** **  * **  * ******** ***

WT         1321 CAGGGAGAGGAGTTTGTGTGCCTCAAATCTATTATTTTGCTTAATTCTGGAGTGTACACA
SYN-opt    1321 CAGGGGGAAGAATTCGTGTGTCTTAAGTCCATCATCCTACTTAACTCCGGGGTGTATACC
                ***** ** ** ** ***** ** ** ** ** **  * ***** ** ** ***** **

WT         1381 TTTCTGTCCAGCACCCTGAAGTCTCTGGAAGAGAAGGACCATATCCACCGAGTCCTGGAC
SYN-opt    1381 TTCCTGAGTAGTACACTGAAATCCCTGGAGGAAAAAGATCACATTCATCGCGTTCTGGAT
                ** ***   ** ** ***** ** ***** ** ** ** ** ** ** ** ** *****

WT         1441 AAGATCACAGACACTTTGATCCACCTGATGGCCAAGGCAGGCCTGACCCTGCAGCAGCAG
SYN-opt    1441 AAAATTACCGATACGCTAATTCATCTGATGGCCAAAGCGGGACTGACACTGCAGCAGCAG
                ** ** ** ** **  * ** ** *********** ** ** ***** ************

WT         1501 CACCAGCGGCTGGCCCAGCTCCTCCTCATCCTCTCCCACATCAGGCACATGAGTAACAAA
SYN-opt    1501 CATCAGAGACTGGCCCAGCTTCTTCTTATTCTTAGTCATATTCGGCATATGTCTAATAAG
                ** *** * *********** ** ** ** **    ** **  **** ***   *** **

WT         1561 GGCATGGAGCATCTGTACAGCATGAAGTGCAAGAACGTGGTGCCCCTCTATGACCTGCTG
SYN-opt    1561 GGAATGGAACACCTGTATAGTATGAAATGTAAAAATGTGGTGCCACTTTACGATCTGCTG
                ** ***** ** ***** ** ***** ** ** ** ******** ** ** ** ******

WT         1621 CTGGAGATGCTGGACGCCCACCGCCTACATGCGCCCACTAGCCGTGGAGGGGCATCCGTG
SYN-opt    1621 CTGGAAATGCTGGATGCCCATAGACTACACGCTCCAACGAGTCGTGGGGGAGCGAGTGTG
                ***** ******** *****  * ***** ** ** ** ** ***** ** **    ***

WT         1681 GAGGAGACGGACCAAAGCCACTTGGCCACTGCGGGCTCTACTTCATCGCATTCCTTGCAA
SYN-opt    1681 GAAGAAACTGATCAAAGTCATCTAGCCACGGCTGGATCCACGTCGAGTCACAGTCTACAA
                ** ** ** ** ***** **  * ***** ** ** ** ** **    **     * ***

WT         1741 AAGTATTACATCACGGGGGAGGCAGAGGGTTTCCCTGCCACGGTCTGA
SYN-opt    1741 AAATACTATATTACTGGAGAAGCGGAAGGGTTTCCCGCCACCGTTTGA
                ** ** ** ** ** ** ** ** ** ** ** ** ***** ** ***
```

Supplementary figure 1: Codon usage optimization of ERα coding sequence by synonymous mutations. (A), Table of amino acid-normalized frequency of codon usage in the two functional gene sets, "proliferation" for "M phase of mitotic cell cycle" and "differentiation" for "pattern specification process" from the study of Gingold et al., (2014) with the corresponding codon usage frequency for ERα gene. (B), Alignment of nucleotide sequences of wild type (WT) and codon usage optimized (SYN-op) ERα. For optimization of codon usage in ERα coding sequence, we chose a codon for each amino acid whose frequency of use in genes specifically expressed in "proliferation" approximates the frequency of the original codon observed in genes specifically expressed in "differentiation". Codons with a similar frequency of use between "proliferation" and "differentiation" were maintained.

A

	Regulatory Element	Binding Factors	strand	Position on sequence	Mismatches
ER WT	Zic-BS1	Zic1; Zic2	+	113-124	1
	Sp1	Sp1	-	1527-1519	0
	Site 2	AP2	+	200-209	0
	NF-kappa B 1	heterodimers p50/RelA; p50/c-Rel; and homodimers of p50 (KBF-1) and RelA	+	1775-1767	0
			-	293-285	0
	Site 5	TF-LF2	-	381-365	3
ER SYN opt	NF-kappa B 1	heterodimers p50/RelA; p50/c-Rel; and homodimers of p50 (KBF-1) and RelA	-	1776-1768	0
	NFkappaB BS	NFkappaB	+	1397-1405	0

	Promoter/Enhancer	Position					
ER WT	No Promoters or Enhancer were predicted						
ER SYN opt	TATA box	821	LDF:	+2.737	TATA box at	799	+7.462 CATAAAAG

B

ER WT **ER SYN opt**

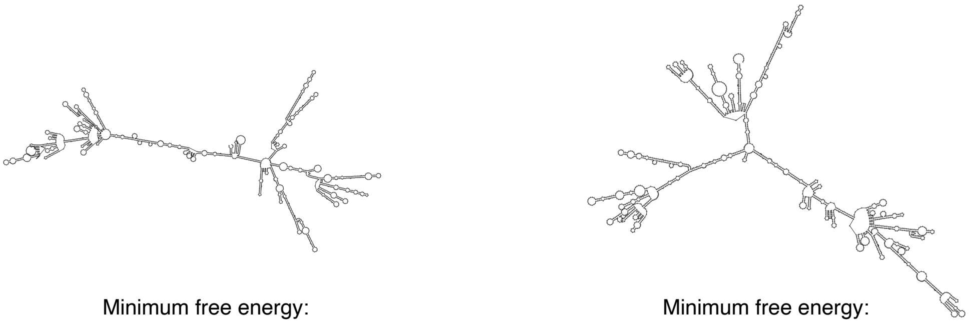

Minimum free energy: **-678.50 kcal/mol**

Minimum free energy: **-544.40 kcal/mol**

Supplementary figure 2: Regulatory elements, promoter/enhancer and secondary mRNA structure predictions from ERα WT and ERα SYN opt coding sequences. (A),Regulatory elements recognition and promoter and enhancer prediction were performed with Nsite and FPROM, respectively (B), Prediction of the optimal secondary structures of mRNA was performed with RNAfolder. Minimal free energies of the secondary structures are indicated.

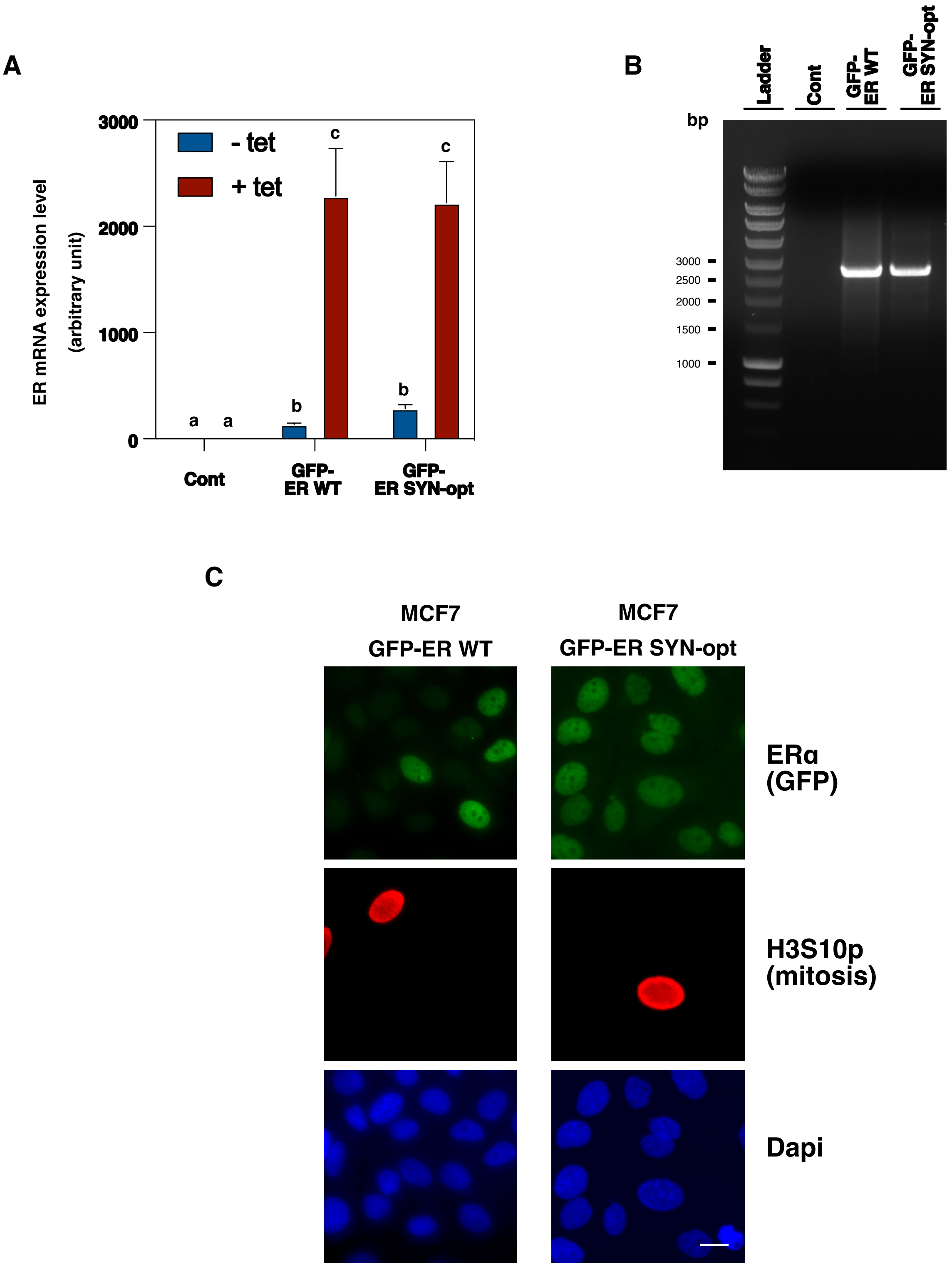

Supplementary figure 3: GFP-ERα mRNA and protein expression in control, GFP-ERα WT and GFP-ERα SYN-op MCF7 subclones. (A), MCF7 subclones were treated or not with tetracycline during 48 h and GFP-ERα mRNA expression was measured by quantitative RT-PCR and then normalized to TBP expression. Primers used to amplify GFP-ERα cDNAs was located in GFP coding region. Results are the means +/- SEM. Columns with different superscripts differ significantly (n=3-6; p<0,05). B), The entire coding region of GFP-ERα cDNAs was amplified by RT-PCR using appropriated primers. Ladder is shown on the right side. (C), GFP-ERα WT and GFP-ERα SYN-opt MCF7 subclones were treated 48 h with tetracycline. Immunofluorescence of H3S10 phosphorylation and GFP fluorescence were measured. Nuclei were stained with dapi. H3S10 phosphorylation identifies cells in mitosis. Scale bar = 10 μm.

Target gene	Forward primer	Reverse Primer
GREB1	GAGGATGTGGAGTGGAGAC	CAGTACCTCAAAGACCTG
CXCL12	CACCATTGAGAGGTCGGAAG	AATGAGACCCGTCTTTGCAG
TFF1	ACCATGGAGAACAAGGTAA	CCGAGCTCTGGGACTAATCA
PGR	GTGCCTATCCTGCCTCTCAATC	CCCGCCGTCGTAACTTTGG
AREG	GTATTTTCACTTTCCGTCTTG	CCTGGGTATATTGTCGATTCA
EGR3	CCTGACAATCTGTACCCCGA	AGTTGGAAGGGGAGTCAAG
TBP	TGCACAGGAGCCAAGAGTGAA	CACATCACAGCTCCCCACCA
BGH region	GTTTAAACCCGCTGATCAGCC	AGGAAAGGACAGTGGGAGTG
Luciferase	TTGTGGATCTGGATACCGGG	AGCCACCTGATAGCCTTTGT
GFP	GGTGAACTTCAAGATCCGCC	CTTGTACAGCTCGTCCATGC

Supplementary Table 1 : List of Primers used to detect target genes by RT-qPCR.
